# Pro-survival responses to the dual inhibition of anti-apoptotic Bcl-2 family proteins and mTOR-mediated signaling in hypoxic colorectal carcinoma cells

**DOI:** 10.1186/s12885-016-2600-y

**Published:** 2016-07-26

**Authors:** Karianne Risberg, Kathrine Røe Redalen, Linda Sønstevold, Tonje Bjørnetrø, Janne Sølvernes, Anne Hansen Ree

**Affiliations:** 1 Department of Oncology, Akershus University Hospital, 1478 Lørenskog, Norway; 2 Institute of Clinical Molecular Biology, Akershus University Hospital, 1478 Lørenskog, Norway; 3 Institute of Clinical Medicine, University of Oslo, 0318 Oslo, Norway

**Keywords:** ABT-737, Apoptosis, AZD8055, Colorectal cancer, Hypoxia, Kinase activity, *KRAS*, Mcl-1, *PIK3CA*

## Abstract

**Background:**

The use of targeted agents to impel dual inhibition of anti-apoptotic mechanisms and mTOR-mediated pro-survival signaling in colorectal carcinoma (CRC) cell lines with *KRAS* or *BRAF* mutation has been shown to induce apoptosis, a timely result given CRC entities harboring such mutations are in need of new therapies. Since CRC comprises heterogeneous tumors with predominant hypoxic components, we investigated effects of an inhibitor of anti-apoptotic Bcl-2 family proteins (ABT-737) in combination with an mTOR inhibitor (AZD8055)—collectively referred to as combo-Rx, in hypoxic CRC cell lines.

**Methods:**

Cell viability measures, expression of proteins implicated in apoptosis and MAPK/PI3K-AKT/mTOR pathway signaling, and profiling of composite kinase activities were undertaken in a panel of 14 cell lines.

**Results:**

In hypoxic conditions, combo-Rx suppressed viability of 13 of the cell lines, albeit ABT-737 did not significantly potentiate the inhibitory effect of single-agent AZD8055 in six of the models. Hypoxic *KRAS/PIK3CA*-mutant HCT-116 and HCT-15 cell lines (both with low endogenous expression of the anti-apoptotic Mcl-1 protein and showing augmented inhibition of viability following the addition of ABT-737 to AZD8055) responded to combo-Rx by induction of apoptosis but with the simultaneous strong Mcl-1 up-regulation and activation of MAPK/PI3K-conducted signaling. In contrast, in hypoxic *KRAS*-mutant LoVo (devoid of *PIK3CA* mutation), *BRAF/PIK3CA*-mutant RKO, and wild-type Colo320DM cell lines (all with high endogenous Mcl-1 expression and being resistant to the additional effect of ABT-737 to AZD8055), combo-Rx did not elicit apoptotic or pro-survival responses.

**Conclusions:**

The concurrent inhibition of anti-apoptotic proteins and mTOR-mediated signaling in hypoxic *KRAS/PIK3CA*-mutant CRC cell lines resulted in pro-survival responses in parallel with the intended anti-proliferative effects, a finding that should be of note if considering combinatory targeting of multiple pathways in this CRC entity.

**Electronic supplementary material:**

The online version of this article (doi:10.1186/s12885-016-2600-y) contains supplementary material, which is available to authorized users.

## Background

Colorectal carcinoma (CRC), like most solid malignancies, comprises heterogeneous tumors with predominant hypoxic components. The adaptive tissue responses to hypoxic stress involve increased resistance to apoptosis (programmed cell death) as well as altered DNA damage repair and mutation rates, and thereby genomic instability [1–3], ultimately leading to compromised efficacy of DNA-damaging therapies (chemotherapy and radiation). Moreover, mutations in genes such as *KRAS*, *BRAF*, and *PIK3CA* commonly result in constitutive activation of cellular signaling mediated by mitogen-activated protein kinases (MAPK) and phosphatidylinositol 3-kinase–protein kinase B (PI3K–AKT) [4, 5]. These pathways converge at the mechanistic target of rapamycin (mTOR), which regulates cell growth and survival [6] and makes the mTOR complex an attractive target for CRC therapy. Consequently, a number of mTOR inhibitors have entered clinical trials.

There is however evidence of crosstalk between the mTOR-conducted signaling and other signaling pathways which will allow tumor cells to escape mTOR-inhibitory therapy [7, 8]. Targeting of multiple pathways has therefore been considered. Recent findings showed that the combination of the mTOR inhibitor AZD8055 with ABT-263, an inducer of apoptosis, promoted cell death in CRC cell lines with *KRAS* or *BRAF* mutation [9], a timely result given CRC entities harboring these mutations are refractory to current targeted therapies. ABT-263 and its structurally related compound ABT-737 are potent inhibitors of the anti-apoptotic proteins Bcl-2, Bcl-xL, and Bcl-w, but not of Mcl-1, and induce apoptosis in cancer cells [10, 11]. Overexpression of Mcl-1 is associated with resistance to ABT-737, and inhibition of Mcl-1 has proven to sensitize cancer cells to ABT-737 [12–14]. Interestingly, hypoxia has been shown to promote ABT-737-mediated apoptotic cell death in small-cell lung carcinoma, CRC, and hematologic cell lines via down-regulation of Mcl-1 [15–17].

Since no information is available regarding the concurrent inhibition of anti-apoptotic proteins and mTOR-mediated pro-survival signaling under CRC tumor hypoxia, we investigated response to treatment with ABT-737 and AZD8055, in this report referred to as combo-Rx, in a panel of hypoxic CRC cell lines harboring various typical mutations.

## Methods

### Cell lines, culture conditions, and reagents

Fourteen human CRC cell lines (kindly provided by Prof. Kjersti Flatmark, Oslo University Hospital, Oslo, Norway or purchased from the American Type Culture Collection, Manassas, VA, USA) were first determined for mutations in *KRAS*, *BRAF*, and *PIK3CA* by Ion Torrent PGM™ sequencing, and mutation profiles were in agreement to already published data [18–20]. All cell lines except Caco-2 were kept in RPMI 1640 medium (Sigma-Aldrich, St. Louis, MO, USA) supplemented with 10 % fetal bovine serum (Gibco by Life Technologies, Grand Island, NY, USA) and 2 mM L-glutamine (GE Healthcare, PAA Laboratories, Pashing, Austria). The Caco-2 cells were kept in DMEM medium (Sigma-Aldrich) containing 15 % serum. The cell lines were routinely tested and found free of mycoplasma infection. For all assays, cells were seeded and left to adhere overnight to reach exponential growth at start of experiments. Cells were incubated under normoxic (21 % O_2_) or hypoxic (0.2 % O_2_) conditions, the latter obtained using the hypoxic chamber Invivo2 300 (Ruskinn Technologies, Leeds, UK). The mTOR inhibitor AZD8055, the PI3K/mTOR inhibitor BEZ235, the Bcl-2 family protein inhibitor ABT-737, and the pan-caspase inhibitor Z-VAD (all by Selleckchem.com, SMS-gruppen, Rungsted, Denmark) were dissolved in dimethyl sulfoxide (Sigma-Aldrich). Control cells received the vehicle.

### Cell viability assay

Depending on the cell line, 12,000-20,000 cells were seeded per well in 96-well Costar plates (Corning Incorporated, Corning, NY, USA). Cells were given ABT-737 or AZD8055, separately or combined, in increasing concentrations (0.10-10 μM; combo-Rx designates 10 μM of both compounds), the combination of ABT-737 and BEZ235 (10 μM of both compounds), or vehicle. When expedient, the cells were pre-treated for 45 min with Z-VAD (20 or 50 μM). Cell viability was determined after 24 or 72 h by adding CellTiter 96^®^AQ_ueous_ One Solution Reagent according to the manufacturer’s instructions (the MTS assay; Promega, Madison, WI, USA). Absorbance was measured using Varioscan (Thermo Electron, Waltham, MA, USA). Values were corrected for background absorbance, and values for treated cells are reported as percentage cell viability to corresponding control cell values. Presented results are from between three and seven independent experiments, each plated at least in triplicate.

### Western blot analysis

Cells were seeded in Nuncleon T25 flasks (Thermo Fisher Scientific, Roskilde, Denmark) and were treated as indicated, and protein lysates from both floating and adherent cells were harvested as previously described [21]. Equal amounts of protein (20 μg) were separated by NuPAGEBis-Tris (Novex by Life Technologies, Carlsbad, CA, USA), transferred by electrophoresis to Immobilon® membrane (Millipore Corporation, Billerica, MA, USA), and probed with antibodies against hypoxia-inducible factor type 1α (HIF-1α; BD Transduction Laboratories, Franklin Lakes, NJ, USA) and carbonic anhydrase IX (CAIX; kindly provided by Prof. Silvia Pastorekova, Slovak Academy of Sciences, Bratislava, Slovak Republic), and against Mcl-1, Bcl-2, Bcl-xL, caspase-3, mitogen-activated protein kinase3/1 (ERK1/2), p-ERK1/2(Thr202/Tyr204), AKT, p-AKT(Ser473), ribosomal protein S6 kinase beta-2 (S6), and p-S6(Ser235/236) (Cell Signaling Technology, La Jolla, CA, USA). Anti-α-tubulin (Calbiochem/EMD Chemicals Inc., San Diego, CA, USA) and Amido Black (Sigma-Aldrich) total protein staining were used as loading controls. Secondary antibodies were from Dako Denmark AS (Glostrup, Denmark). Peroxidase activity was visualized using SuperSignal West Dura Extended Substrate (Thermo Scientific, Rockford, IL, USA). Sufficient amount of lysate from each sample was prepared to run three gels. The parallel blotting membranes were considered identical, and different proteins were visualized on different membranes for practicality. All Western blot experiments were performed as three biological replicates.

### RNA interference

Mcl-1 expression was inhibited using short hairpin (sh)RNA (clone ID NM_021960.3-953s1c1; Sigma-Aldrich), and control cells were generated using non-target sequence (product number shc002v; Sigma-Aldrich). The manufacturer’s instructions were followed apart from extending the lentiviral incubation period to 48 h.

### Microscopy

Cells were seeded in Nuncleon T25 flasks and treated as indicated for up to 72 h. When expedient, the cells were pre-treated for 45 min with Z-VAD. Phase-contrast images were processed at the start of experiment and further after 24, 48, and 72 h by Olympus IX81 (Olympus Europa Holding GmbH, Hamburg, Germany).

### Kinase activity profiling

The Tyrosine Kinase PamChip® Array technology (PamGene International B.V., ‘s-Hertogenbosch, The Netherlands) enables profiling of composite tissue kinase activities [4]. The array contains peptides that are kinase substrates and consisting of 13 or 14 amino acids with tyrosine residues for phosphorylation. Protein lysates used for Western blot analysis were also incubated on the arrays for kinase activity profiling. Substrate phosphorylation intensities were measured using the Evolve software (PamGene International B.V.). Applying BioNavigator software (PamGene International B.V.), endpoint signal intensities generated from bound fluorescent anti-phosphotyrosine antibody were converted to numerical values. The primary array data are available in the ArrayExpress data repository (http://www.ebi.ac.uk/arrayexpress/experiments/E-MTAB-3870/) by accession number E-MTAB-3870. Background signals were subtracted, and negative signal intensities were managed by subtracting the 1 % quantile of all data and setting the remaining signal intensities less than 1 to the value of 1. Data were log_2_-transformed before mean signal intensity of three replicates that were analyzed for each experimental condition was calculated for each peptide substrate. The resulting data from each type of treatment was compared to the relevant control (vehicle-treated cells) for assessment of increase or decrease in substrate phosphorylation level. Substrates associated with PI3K-AKT and/or MAPK pathways were retrieved from PathCards (http://pathcards.genecards.org/), applying the super-pathway definitions ‘PI3K-AKT signaling pathway’ and ‘MAPK signaling pathway’.

### Statistical analysis

Differences between groups were analyzed using two-tailed Student’s *t*-test. *p*-values less than 0.05 were considered statistically significant. In the assessment of combination effects on cell viability, we chose to evade calculations based on median-effect equation for multiple drug interactions because the ABT-737 single-agent effects did not appear with typical dose-response curves.

## Results

### Inhibition of anti-apoptotic proteins or mTOR-mediated signaling—cell viability

First, individual effects of the Bcl-2 family protein inhibitor ABT-737 and the mTOR inhibitor AZD8055 on cell viability under hypoxia and normoxia at 24 h were examined (Additional file 1: Fig. S1a). Incubation of HCT-116, RKO, HT-29, and Colo320DM cell lines with increasing concentrations of ABT-737 (0.10–10 μM) had modest effects. In contrast, differential sensitivity was observed with AZD8055 (0.10–10 μM), with HT-29 cells being refractory and HCT-116, RKO, and Colo320DM cells displaying suppressed cell viability in the order of 20–60 % depending on the oxygenation status. The intended cellular response to hypoxia was confirmed by the time-dependent induction of HIF-1α and its target CAIX in HCT-116 cells (Additional file 1: Fig. S1b).

Since Faber and co-workers found that the combination of ABT-263, a structurally related compound to ABT-737, with AZD8055 at concentrations of 50–500 nM for 72 h caused apoptosis in CRC cell lines with *KRAS* or *BRAF* mutation [9], we next investigated inhibitory effects on cell viability when combining AZD8055 at 0.10 μM with ABT-737 for 72 h. For seven tested cell lines, ABT-737 potentiated AZD8055 under hypoxia in only one of five mutant ones (of which three and two had *KRAS* and *BRAF* mutation, respectively; one of each had also *PIK3CA* mutation) but in both of the wild-type models (Additional file 2: Tables S1a and S1b).

### Combo-Rx and hypoxic cell viability

Based on the two initial sets of results, the highest tested concentration (10 μM) of both compounds was chosen for further experiments. First, we investigated viability in 14 CRC cell lines under normoxic conditions (Additional file 2: Table S2). The addition of ABT-737 significantly potentiated inhibitory effects of AZD8055 in four of six *KRAS*-mutant and one of three wild-type cell lines but in none of five *BRAF*-mutant models. The findings in the two first-mentioned groups but not in *BRAF*-mutant cell lines were generally in agreement with previously reported data [9].

**Fig. 1:**
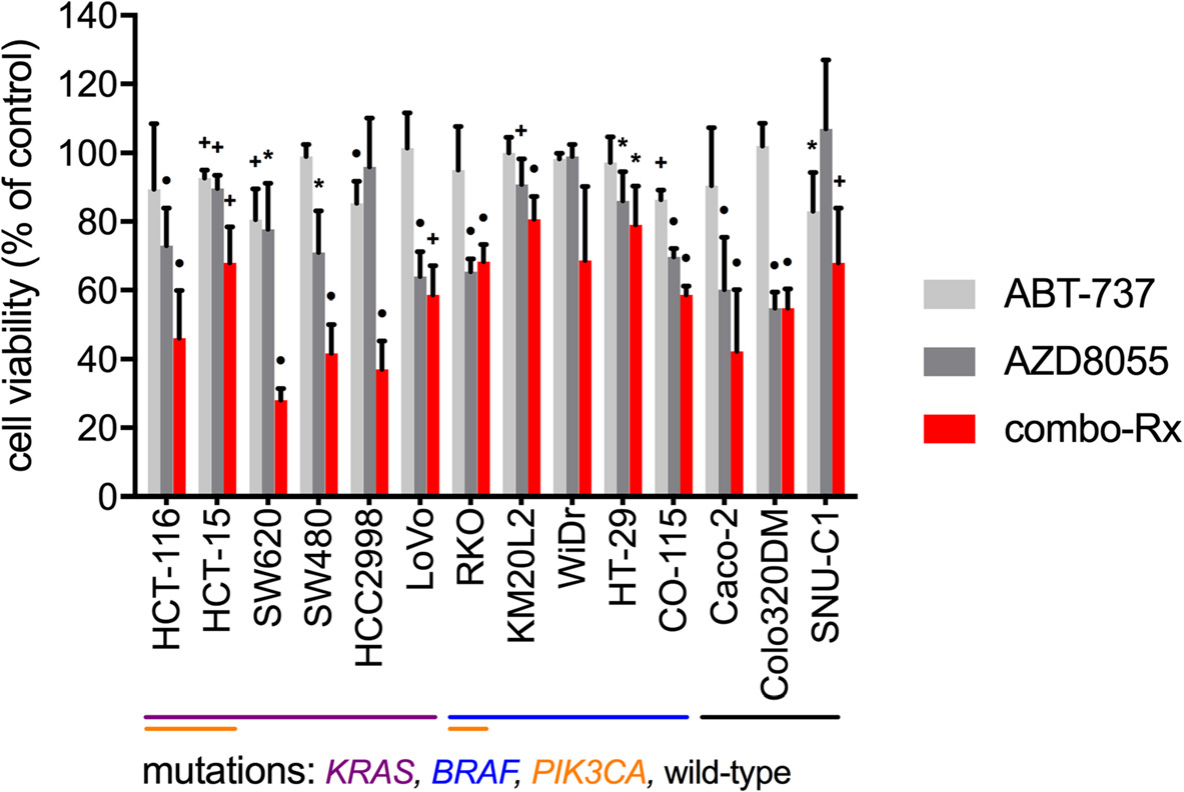
Cell viability in hypoxic colorectal carcinoma (CRC) cell lines. Fourteen CRC cell lines were treated for 24 h with ABT-737 (inhibitor of anti-apoptotic Bcl-2 family proteins; 10 μM), AZD8055 (mTOR inhibitor; 10 μM), or combo-Rx (10 μM of both compounds in combination) under hypoxic conditions. Cell viability (measured by the MTS assay) value for each condition relative to the corresponding control cell value is shown as mean ± SD. Statistically significant changes are indicated (asterisk, *p* < 0.05; cross, *p* < 0.01; circle, *p* < 0.001)

Specifically, we were interested in elucidating inhibitory effects of combo-Rx under hypoxic conditions in the 14 cell lines (Fig. 1). Again, single-agent ABT-737 caused limited decrease in cell viability (median decline of 6.2 % (range,−2.0 to 19 %) across cell lines), which was significant in five cell lines only. Incubation with AZD8055 (by itself resulting in a median decline of 25 % (range,−7.0 to 45 %) across the cell lines) or combo-Rx significantly suppressed cell viability in 11 and 13 of the 14 cell lines, respectively. However, as shown in Table 1, in six of the hypoxic cell lines (one of six *KRAS*-mutant, three of five *BRAF*-mutant, and two of three wild-type ones), ABT-737 did not significantly potentiate the inhibitory effect of single-agent AZD8055. Three of the 14 cell lines, two sensitive and one resistant to the additional effect of ABT-737, had *PIK3CA* mutation. Similarly, treatment with the PI3K/mTOR inhibitor BEZ235, which has demonstrated anti-proliferative effects in various models [22, 23], caused a median decline of 22 % (range, 3.5 to 47 %) in cell viability across hypoxic cell lines, which in five of 12 cell lines was not significantly potentiated by ABT-737 (Additional file 2: Tables S3a and S3b).

**Table 1:** Cell viability of hypoxic human colorectal carcinoma cell lines

Cell line	Mutations	ABT-737	AZD8055	combo-Rx	*p*-value
HCT-116	*KRAS, PIK3CA*	89.4 ± 19.1	73.0 ± 10.9	46.1 ± 13.7	0.0016
HCT-15	*KRAS, PIK3CA*	92.7 ± 2.31	89.7 ± 3.79	68.0 ± 10.4	0.028
SW620	*KRAS*	80.6 ± 8.91	77.8 ± 13.4	28.0 ± 3.46	<0.001
SW480	*KRAS*	99.0 ± 3.46	71.0 ± 12.1	41.7 ± 8.39	0.026
HCC2998	*KRAS*	85.3 ± 6.41	96.0 ± 14.2	37.0 ± 8.37	<0.001
LoVo	*KRAS*	101 ± 10.3	64.0 ± 7.21	58.7 ± 8.50	0.45
RKO	*BRAF, PIK3CA*	95.0 ± 12.8	65.3 ± 3.79	68.3 ± 5.03	0.46
KM20L2	*BRAF*	100 ± 4.62	90.9 ± 7.38	80.7 ± 6.63	0.019
WiDr	*BRAF*	98.3 ± 1.71	99.0 ± 3.46	68.7 ± 21.6	0.074
HT-29	*BRAF*	97.3 ± 7.48	86.0 ± 8.54	79.0 ± 11.4	0.44
CO-115	*BRAF*	86.3 ± 2.89	69.7 ± 2.52	58.7 ± 2.52	0.0059
Caco-2	wild-type	90.4 ± 17.0	60.1 ± 15.3	42.3 ± 17.9	0.068
Colo320DM	wild-type	102 ± 6.73	54.9 ± 4.56	54.9 ± 5.46	1.0
SNU-C1	wild-type	83.0 ± 11.4	107 ± 20.0	68.0 ± 15.9	0.023

From between three and seven independent experiments, each at least with triplicate setups, mean ± SD cell viability values were calculated in percentage of values from the corresponding controls (vehicle-treated cells). Treatments (24-h incubations): ABT-737 (inhibitor of anti-apoptotic Bcl-2 family proteins; 10 μM), AZD8055 (mTOR inhibitor; 10 μM), combo-Rx (10 μM of both compounds in combination). Difference in values from cells given AZD8055 or combo-Rx was compared by two-tailed Student’s *t*-test. Mutation status is indicated for each cell line

### Combo-Rx—Mcl-1 and apoptotic response

**Fig. 2:**
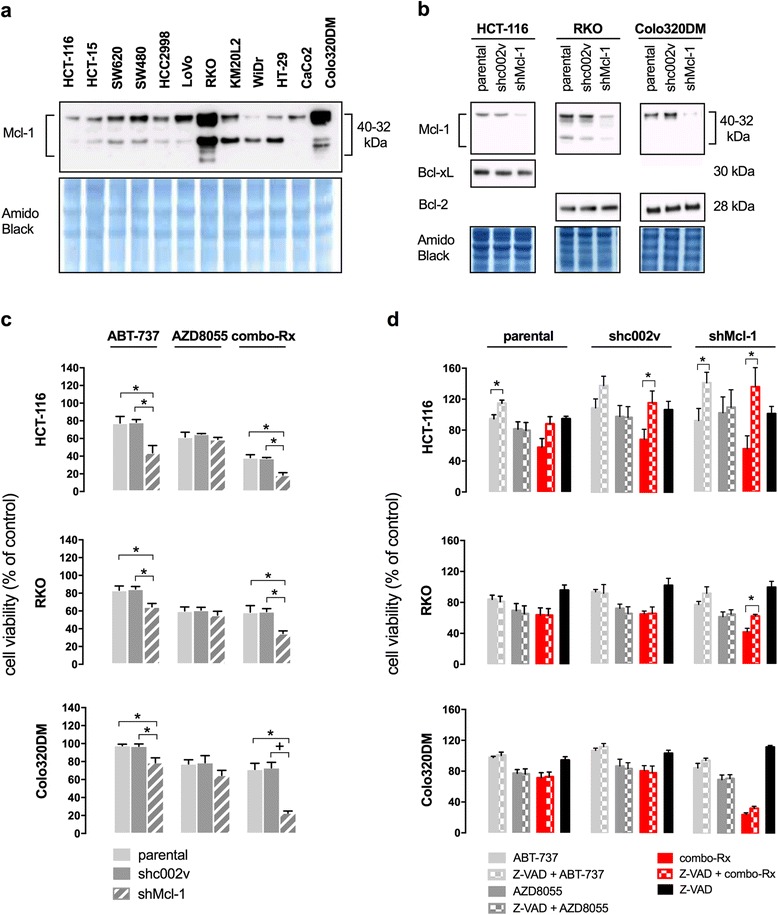
The anti-apoptotic Mcl-1 protein and hypoxic cell viability. **a** Expression of Mcl-1 in a panel of human colorectal carcinoma cell lines, as illustrated by Western blot analysis. The experiment was performed twice. **b** Three parental cell lines were treated with short hairpin (sh)RNA of Mcl-1 and a non-target control sequence (shc002v), and expression of anti-apoptotic proteins was examined by Western blot analysis. The experiment was performed at least three times for each of the cell line groups. **c** The parental and the shc002v and shMcl-1 versions of the three cell lines were treated for 24 h with ABT-737 (inhibitor of anti-apoptotic Bcl-2 family proteins; 10 μM), AZD8055 (mTOR inhibitor; 10 μM), or combo-Rx (10 μM of both compounds in combination) under hypoxic conditions. Cell viability (measured by the MTS assay) values for treated cells relative to the corresponding controls (mean ± SD) are shown. Statistically significant differences between cell line versions are indicated (asterisk, *p* < 0.05; cross, *p* < 0.01). **d** The same cell line entities were treated for 24 h under hypoxic conditions with ABT-737, AZD8055, or combo-Rx alone or following pre-treatment for 45 min with the pan-caspase inhibitor Z-VAD (20 μM), as indicated. Cell viability values relative to the corresponding control values are shown as mean ± SD. Statistically significant differences between treatment groups are indicated (asterisk, *p* < 0.05; cross, *p* < 0.01)

Further focusing on the finding that ABT-737 was unable to significantly potentiate the effect of AZD8055 on cell viability in approximately half of hypoxic cell lines investigated and moreover, since previous studies have shown that suppression of the anti-apoptotic Mcl-1 protein sensitizes cells to ABT-737 [12–14], Mcl-1 was knocked down in the *KRAS/PIK3CA*-mutant HCT-116 cell line (with low endogenous Mcl-1 expression and being sensitive to the additional effect of ABT-737) and the *BRAF/PIK3CA*-mutant RKO and wild-type Colo320DM cell lines (both with high endogenous Mcl-1 expression and being resistant to the additional effect of ABT-737) (Fig. 2a). Specific knockdown of Mcl-1, though not complete, and not of other anti-apoptotic proteins was confirmed (Fig. 2b). As shown in Fig. 2c, Mcl-1 knockdown significantly sensitized for inhibitory cell viability effects by ABT-737 and combo-Rx in all three hypoxic cell lines. Next, when the Mcl-1-repressed cell lines were pre-treated with the pan-caspase inhibitor Z-VAD (20 μM) in order to examine to which extent apoptosis might account for responses, the inhibitory cell viability effect was completely abolished in hypoxic shMcl-1 HCT-116 cells given ABT-737 or combo-Rx and partly counteracted by combo-Rx in hypoxic shMcl-1 RKO cells (Fig. 2d). On increasing the Z-VAD concentration to 50 μM, no further regulatory effects were seen in the Mcl-1-repressed RKO and Colo320DM cell lines (data not shown). These results do not exclude the possibility that the low levels of remaining Mcl-1 protein were able to protect these cell lines to inhibitory effects of ABT-737, as complete knockdown of Mcl-1 was not obtained (Fig. 2b). Nevertheless, the data collectively indicated two different modes of hypoxic cell viability response, one clearly involving apoptosis (in HCT-116 cells) and another not.

To explore the notion of non-apoptotic mechanisms of combo-Rx effects in RKO and Colo320DM cell lines, the three hypoxic cell lines were treated for 72 h and inspected at 24-h intervals (Additional file 3: Fig. S2). In HCT-116 cells, a mixture of pycnotic cells indicating apoptosis [24] and still surface-attached cells was observed at 24 h. This particular finding of a dual phenotype remained for the entire incubation period of 72 h (data not shown). In the Colo320DM and RKO cell lines, combo-Rx caused decrease in cell confluence, indicating growth-inhibitory effects, but essentially few pycnotic cells after 48 and 72 h, respectively. The Colo320DM cells did not tolerate the combo-Rx drug concentrations in hypoxia for 72 h. In neither of the two cell lines, the growth inhibition following combo-Rx was abolished by Z-VAD (data not shown), again arguing against the involvement of apoptosis.

### Combo-Rx—activation of hypoxic pro-survival signaling

**Fig. 3:**
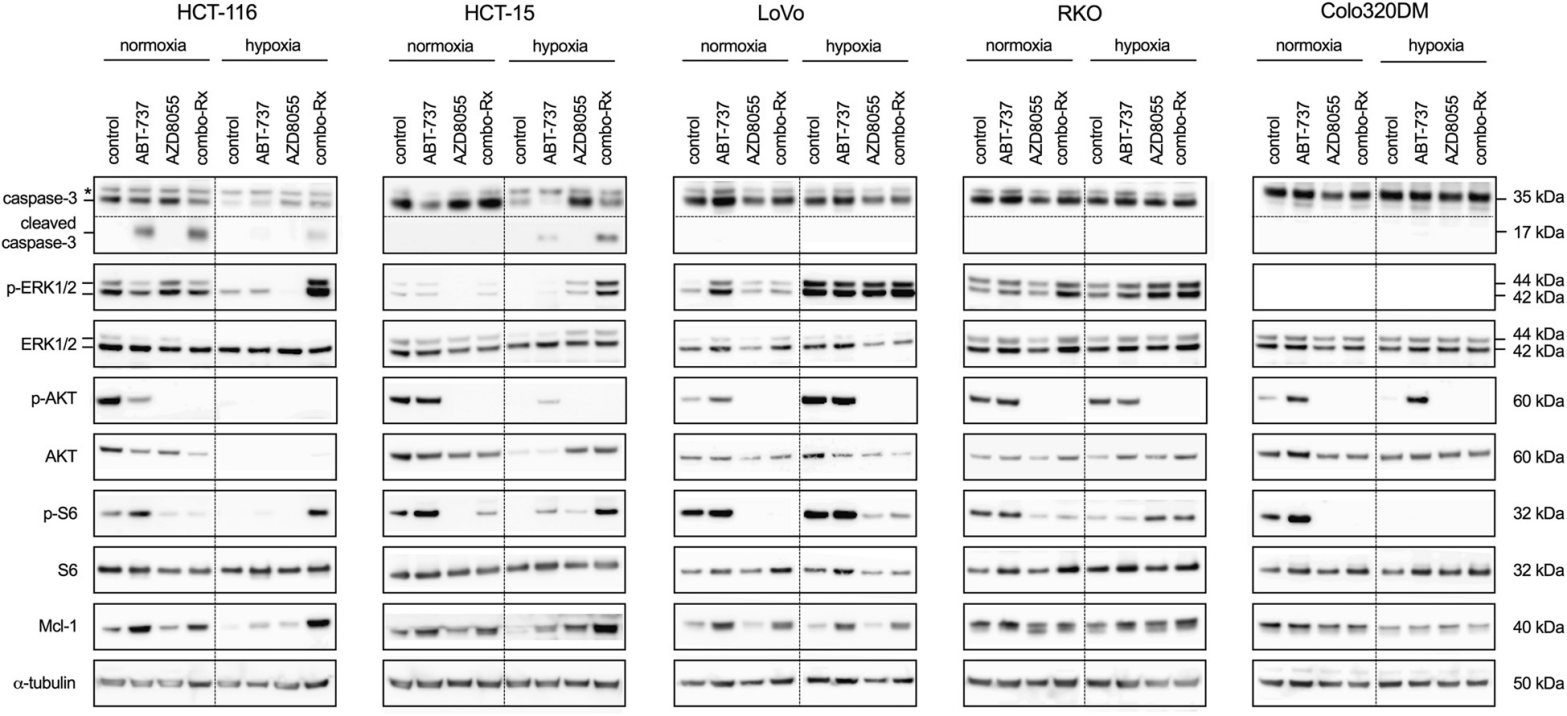
Expression of proteins implicated in apoptosis and MAPK/PI3K-AKT/mTOR pathway signaling. The five cell lines were treated for 24 h with ABT-737 (inhibitor of anti-apoptotic Bcl-2 family proteins; 10 μM), AZD8055 (mTOR inhibitor; 10 μM), or combo-Rx (10 μM of both compounds in combination), and protein expression was examined by Western blot analysis. Cleaved capspase-3 (17 kDa) is a marker for apoptosis. *, unspecific band. ERK1/2, AKT, and S6 are mediator proteins of MAPK, PI3K-AKT, and mTOR signaling. Mcl-1 is an anti-apoptotic Bcl-2 family protein. Abbreviation: p, phosphorylation. For transparency, the dotted lines indicate where membranes had been cut as explained in the Methods section. The experiments were performed three times each

Following the observations in hypoxic HCT-116 cells that combo-Rx was effective in inhibiting viability but caused both surface-attached and pycnotic cells, underlying mechanisms for the regulatory effects were investigated. The HCT-116, RKO, and Colo320DM cell lines from the preceding sets of experiments were complemented with HCT-15 cells (*KRAS/PIK3CA*-mutant and sensitive to the additional effect of ABT-737 to ABT8055) and LoVo cells (with high endogenous Mcl-1 expression (Fig. 2a) and the only *KRAS*-mutant model that was resistant to combo-Rx for viability). The five cell lines were assessed for induction of apoptosis, as indicated by cleavage of caspase-3, and for pro-survival signaling by phosphorylation of ERK1/2 (p-ERK1/2) of the MAPK pathway, of AKT (p-AKT(Ser473)) of the PI3K-AKT pathway, and of S6 (p-S6) down-stream of mTOR, and by expression of the anti-apoptotic protein Mcl-1 (Fig. 3).

Of initial note, 24 h of hypoxic incubation in itself (i.e.*,* of control cells) inhibited Mcl-1 expression in the majority of the cell lines. Although levels of caspase-3 were also lower under hypoxic compared to normoxic conditions in both cell lines that were sensitive to the additional effect of ABT-737 to ABT8055 (HCT-116 and HCT-15), cleaved caspase-3 was observed after 24 h of hypoxic combo-Rx. As expected, this apoptotic feature could not be detected in the three resistant cell lines (LoVo, RKO, and Colo320DM). Next, under normoxia, ABT-737 treatment caused Mcl-1 up-regulation and p-S6 induction as a consequence, and these responses were counteracted by the addition of AZD8055 (i.e.*,* under combo-Rx compared to ABT-737 treatment alone). These observations were in agreement with previously reported data [9]. Under hypoxia also, Mcl-1 up-regulation by ABT-737 alone was generally seen in all cell lines with mutations (HCT-116, HCT-15, LoVo, and RKO); however, a striking response followed the addition of AZD8055 to ABT-737. The two models that showed potentiation with combo-Rx (HCT-116 and HCT-15; both also with *PIK3CA* mutation) demonstrated a further strong induction of Mcl-1 and p-S6 and also of p-ERK1/2. In contrast, hypoxic combo-Rx did not cause any uniform pattern of regulation of these selected pro-survival factors in the resistant cell lines. Specifically, in the *KRAS*-mutant LoVo cell line (devoid of *PIK3CA* mutation), the addition of AZD8055 counteracted the effects of ABT-737 on Mcl-1 and p-S6, exactly like under normoxia. The hypoxic *BRAF/PIK3CA*-mutant RKO cells showed Mcl-1 and p-S6 responses similar to but not as strong as those of the HCT-116 and HCT-15 models. And in the wild-type Colo320 cell line, hypoxic combo-Rx did not cause Mcl-1 alteration nor was p-S6 detected. In summary, the results highlighted the possibility of dual apoptotic and pro-survival responses in *KRAS/PIK3CA*-mutant CRC cells responding under hypoxic conditions with augmented inhibition of viability to the addition of ABT-737 to AZD8055.

### Combo-Rx—activation of hypoxic kinase signaling

Following the intriguing results that phosphorylation of a discrete number of MAPK/mTOR-signaling mediators was induced by combo-Rx in hypoxic *KRAS/PIK3CA*-mutant cell lines, we applied the Tyrosine Kinase PamChip® Array to assess HCT-116 kinase activities more broadly. The *KRAS*-mutant HCC2998 cell line, which also responded to hypoxic combo-Rx with augmented inhibition of viability but did not harbor *PIK3CA* mutation (Table 1), and the non-responding wild-type Colo320DM cell line, in which p-ERK1/2 and p-S6 had not been detected (Fig. 3), were analyzed for comparison.

**Fig. 4:**
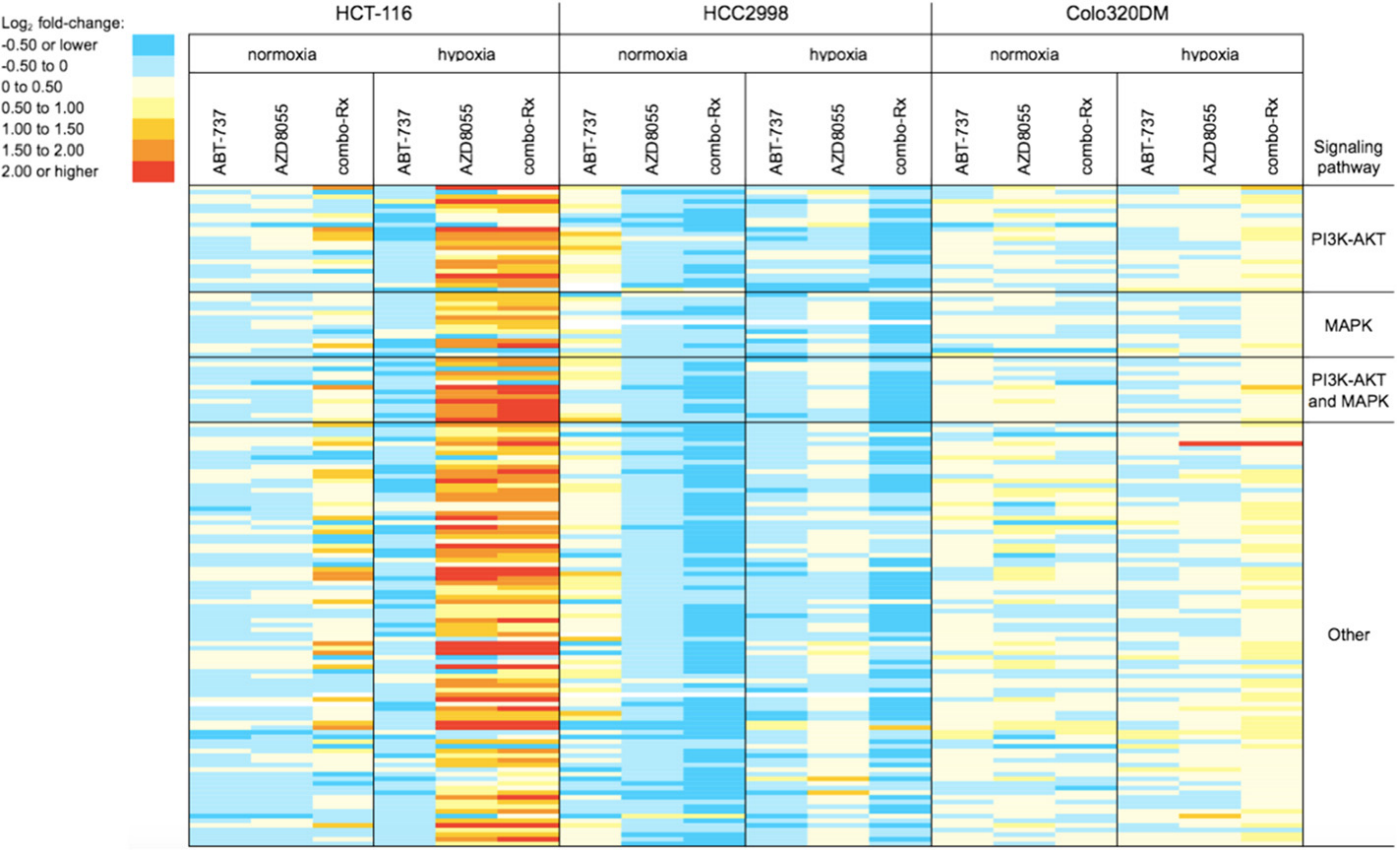
Ex vivo kinase substrate phosphorylation. The color map visualizes normalized log_2_-transformed signal intensities from kinase substrate arrays incubated with lysates from the three cell lines treated for 24 h with ABT-737 (inhibitor of anti-apoptotic Bcl-2 family proteins; 10 μM), AZD8055 (mTOR inhibitor; 10 μM), or combo-Rx (10 μM of both compounds in combination). As quantified and categorized by the color codes, red corresponds to higher and blue to lower substrate phosphorylation levels relative to levels from the corresponding control cells (vehicle-treated). Substrates associated with PI3K-AKT and/or MAPK signaling pathways were retrieved from PathCards (http://pathcards.genecards.org/), applying the super-pathway definitions ‘PI3K-AKT signaling pathway’ and ‘MAPK signaling pathway’. The identity of each peptide substrate, vertically in order from top to bottom, is given in Additional file 4: Table S4

As depicted in Fig. 4 and detailed in Additional file 4: Table S4, ABT-737 inhibited the kinase activity in both hypoxic and normoxic HCT-116 cells. Intriguingly, lysates from hypoxic HCT-116 cells given AZD8055 or combo-Rx generated strong phosphorylation of the majority of the array peptides and not explicitly substrates associated with the MAPK or PI3K-AKT signaling pathways. In particular, peptides representing proteins involved in the angiogenic response to hypoxia (such as PDGFRB, EPOR, ANXA2, CTTN1, MET, PXN, and PECAM1) and MAPK/PI3K-conducted signaling in particular (RASA1, RAF1, PIK3R1, and PDPK1) were highly phosphorylated (log_2_ fold-change >2.0; Additional file 4: Table S4). In normoxic HCT-116 cells, treatment with AZD8055 generally suppressed kinase activity, while combo-Rx caused increased phosphorylation (but by log_2_ enhancement <2.0) of a limited number of substrates. In contrast, kinase activity of HCC2998 cells, whether hypoxic or normoxic, was predominantly repressed by all treatment conditions, while Colo320DM kinase activity was basically unchanged.

## Discussion

A number of recent findings [4, 9, 16, 17] led us to investigate the effects of ABT-737, an inhibitor of anti-apoptotic Bcl-2 family proteins, in combination with the mTOR inhibitor AZD8055 in a panel of 14 hypoxic CRC cell lines. Combo-Rx (i.e.*,* the combination treatment) suppressed viability of 13 of the cell lines, albeit ABT-737 did not significantly potentiate the inhibitory effect of single-agent AZD8055 in six of the models. On further mechanistic investigations, the hypoxic *KRAS/PIK3CA*-mutant HCT-116 and HCT-15 cell lines (both with low endogenous expression of the anti-apoptotic Mcl-1 protein and showing augmented inhibition of viability following the addition of ABT-737 to AZD8055) responded to combo-Rx by induction of apoptosis (as assessed by various experimental approached in the HCT-116 cells) and with the simultaneous strong Mcl-1 up-regulation and activation of MAPK/PI3K-conducted signaling. A ubiquitous activation of hypoxic kinase signaling by combo-Rx was also confirmed in the HCT-116 cells. In contrast, in hypoxic *KRAS*-mutant LoVo, *BRAF/PIK3CA*-mutant RKO, and wild-type Colo320DM cell lines (all with high endogenous Mcl-1 expression and being resistant to the additional effect of ABT-737 to AZD8055), combo-Rx did not elicit apoptotic or pro-survival responses. Collectively, this data revealed complex responses to the concurrent inhibition of anti-apoptotic proteins and mTOR-mediated signaling in hypoxic CRC cell lines, where pro-survival responses were elicited in parallel with the intended anti-proliferative effects in *KRAS/PIK3CA*-mutant entities in particular, a finding that should be of note if considering the combinatory targeting of multiple pathways in CRC treatment.

As recently shown [9], when combined with ABT-263 (structurally related to ABT-737), AZD8055 via the specific suppression of Mcl-1 sensitized CRC cell lines with *KRAS* or *BRAF* mutation to undergo apoptosis, a timely result as such CRC entities are refractory to current targeted therapies. In vitro studies have shown promising treatment effects of both types of agents, but concerns have been raised with regard to lacking therapeutic efficacy of mTOR inhibitors in solid tumors [11, 25]. Both AZD8055 and the anti-apoptotic inhibitors ABT-263 and ABT-737 have reached early-phase clinical trials. However, only a few reports exist on the use of ABT-263 or ABT-737 in hypoxic tumor models [15, 16], and as pointed out by Harrison and co-workers [16], whether Mcl-1 is up- or down-regulated may be cell type- and oxygen concentration-dependent. To our knowledge, little information is currently available on effects of AZD8055 or other mTOR inhibitors under hypoxic conditions, with the exception of BEZ235 which has been shown to sensitize hypoxic breast and prostate cancer cells to radiation [26, 27].

Invariably, CRC comprises heterogeneous tumors with predominant hypoxic components [4], which is important to take into consideration with established as well as novel therapies. The present study showed that combo-Rx significantly suppressed viability of hypoxic CRC cells, but in six of 14 cell lines there was no additional inhibitory effect of ABT-737 to that of single-agent AZD8055. A similar finding was obtained with the combination of ABT-737 and BEZ235 (potentiation was not seen in five of 12 cell lines) and when AZD8055 in a lower concentration of 0.10 μM was given together with ABT-737 (four of seven hypoxic cell lines were resistant to an additional effect of the anti-apoptotic inhibitor). In the five cell lines where mechanistic investigations were undertaken (the *KRAS*-mutant HCT-116, HCT-15, and LoVo models and the *BRAF/PIK3CA*-mutant RKO and wild-type Colo320DM cells), combo-Rx under hypoxic conditions caused dual phenotypic responses in terms of concurrent apoptotic and pro-survival effects in the HCT-116 and HCT-15 cell lines, as demonstrated through the specific examination of the anti-apoptotic Mcl-1 protein, microscopy of cell cultures, and both targeted and comprehensive analysis of kinase signaling. Importantly, both of these cell lines also harbor *PIK3CA* mutation. These findings suggest that CRC cancers with co-occurring *KRAS* and *PIK3CA* mutations, which is not a frequent entity [5], may be particularly susceptible to parallel apoptotic and pro-survival effects with this combination treatment.

The MAPK and PI3K-AKT signaling pathways merge at the mTOR complex, which promotes cell survival through phosphorylation of S6 and the resulting increase in Mcl-1 protein translation [28]. Intriguingly, in hypoxic HCT-116 and HCT-15 cells, combo-Rx strongly increased expression of Mcl-1 and p-S6, which under normoxia and in agreement with previously reported data [9] showed the opposite response following the addition of AZD8055 to ABT-737. The Tyrosine Kinase PamChip® Array approach enabled the investigation of more general kinase activity responses. Using this technology, ABT-737 was shown to repress kinase activities known to be important for CRC survival in hypoxic HCT-116 cells. Under normoxic conditions, ABT-737 treatment has been shown to sensitize CRC and rhabdomyosarcoma cell lines for AZD8055-directed apoptosis [9, 29]. It is therefore notable that in our experimental setups, a number of array substrates associated with MAPK/PI3K-conducted signaling (RASA1, RAF1, PIK3R1, and PDPK1) were phosphorylated by lysates from hypoxic HCT-116 cells given combo-Rx. Moreover, array substrates reflecting proteins that are fundamental in the angiogenic response to hypoxia, particularly PDGFRB, were also highly phosphorylated. In angiogenesis, PDGFR is required for the formation of a functional pericyte coverage of regenerating endothelium within the tumor stroma [30]. Importantly, the *KRAS*-mutant HCC2998 cell line, which was sensitive to combo-Rx for viability but devoid of *PIK3CA* mutation, and the resistant wild-type Colo320DM model, showed repressed or unchanged global kinase activities to the experimental perturbations. Again, the findings indicate a particular susceptibility of *KRAS/PIK3CA*-mutant CRC entities to unfavorable responses to the combined inhibition of anti-apoptotic proteins and mTOR signaling. Of final note, in the context of interpreting the ex vivo kinase substrate data, some important considerations should be kept in mind [4]. One is that hypoxia elicits a multitude of adaptive signaling responses, which as such are challenging to portray, and another is that phosphorylation of each individual substrate on the kinase target array reflects the net result of an extensive network of multiple kinase activities.

## Conclusions

To conclude, under hypoxia, which is an important feature of CRC tumors and a main mechanism of therapy resistance, the apparently rational approach of combining ABT-737 for inhibition of anti-apoptotic proteins with the mTOR inhibitor AZD8055 [9] in CRC cell lines with co-occurring *KRAS* and *PIK3CA* mutations revealed complex responses of pro-survival effects elicited in parallel with apoptosis. Recognizing the obvious limitation that CRC cell lines do not fully reflect the heterogeneity of this disease with regard to mutation profiles or the dynamic nature of tumor oxygenation status, more extensive preclinical studies are required for further elucidation of responses to this kind of combination treatment. Still, from a clinical perspective, and particularly with tumor hypoxia posting a demand for more tailored therapies [31], caution should be practiced on implementation of combinatory strategies.

## Abbreviations

CAIX: Carbonic anhydrase IX; CRC: Colorectal carcinoma; ERK1/2: Mitogen-activated protein kinase 3/1; HIF-1α: Hypoxia-inducible factor type 1α; MAPK: Mitogen-activated protein kinases; mTOR: Mechanistic target of rapamycin; PI3K-AKT: Phosphatidylinositol 3-kinase–protein kinase B; S6: Ribosomal protein S6 kinase beta-2; sh: Short hairpin

## Additional files

Additional file 1: Fig. S1. Cell viability of hypoxic colorectal carcinoma (CRC) cell lines. a. Four CRC cell lines were treated for 24 h with the indicated concentrations of ABT-737 (inhibitor of anti-apoptotic Bcl-2 family proteins) or AZD8055 (mTOR inhibitor) under normoxic or hypoxic culture conditions. Cell viability (measured by the MTS assay) value for each condition relative to the corresponding control cell (vehicle-treated) value is shown as mean ± SD from at least three independent experiments, each plated at least in triplicate. Statistically significant changes are indicated (asterisk, *p* < 0.05; cross, *p* < 0.01; circle, *p* < 0.001). b. Cultures of the CRC cell line were left in the hypoxic chamber at 0.2 % O_2_ and harvested after the indicated time periods. Expression of the hypoxia-inducible factor type 1α (HIF-1α) and its target gene carbonic anhydrase IX (CAIX) was examined by Western blot analysis, with α-tubulin expression as loading control, and with 24 h of normoxia and 4 h of 100 μM CoCl_2_ exposure in normoxia to generate negative and positive biological controls for HIF-1α expression, respectively. (DOCX 369 kb) Additional file 2: Table S1a. Cell viability of normoxic colorectal carcinoma cell lines. From at least three independent experiments, each at least with triplicate setups, mean ± SD cell viability values were calculated in percentage of values from the corresponding controls (vehicle-treated cells). Treatments (72-h incubations): ABT-737 (inhibitor of anti-apoptotic Bcl-2 family proteins; 10 μM), AZD8055 (mTOR inhibitor; 0.10 μM), both compounds in combination. Difference in values from cells given AZD8055 or the combination treatment was compared by two-tailed Student’s *t*-test. Mutation status is indicated for each cell line. **Table S1b.** Cell viability of hypoxic colorectal carcinoma cell lines. From at least three independent experiments, each at least with triplicate setups, mean ± SD cell viability values were calculated in percentage of values from the corresponding controls (vehicle-treated cells). Treatments (72-h incubations): ABT-737 (inhibitor of anti-apoptotic Bcl-2 family proteins; 10 μM), AZD8055 (mTOR inhibitor; 0.10 μM), both compounds in combination. Difference in values from cells given AZD8055 or the combination treatment was compared by two-tailed Student’s *t*-test. Mutation status is indicated for each cell line. **Table S2.** Cell viability of normoxic colorectal carcinoma cell lines. From between three and seven independent experiments, each at least with triplicate setups, mean ± SD cell viability values were calculated in percentage of values from the corresponding controls (vehicle-treated cells). Treatments (24-h incubations): ABT-737 (inhibitor of anti-apoptotic Bcl-2 family proteins; 10 μM), AZD8055 (mTOR inhibitor; 10 μM), both compounds in combination (combo-Rx). Difference in values from cells given AZD8055 or combo-Rx was compared by two-tailed Student’s *t*-test. Mutation status is indicated for each cell line. **Table S3a.** Cell viability of normoxic colorectal carcinoma cell lines. From at least three independent experiments, each at least with triplicate setups, mean ± SD cell viability values were calculated in percentage of values from the corresponding controls (vehicle-treated cells). Treatments (24-h incubations): ABT-737 (inhibitor of anti-apoptotic Bcl-2 family proteins; 10 μM), BEZ235 (PI3K/mTOR inhibitor; 0.10 μM), both compounds in combination. Difference in values from cells given BEZ235 or the combination treatment was compared by two-tailed Student’s *t*-test. Mutation status is indicated for each cell line. **Table S3b.** Cell viability of hypoxic colorectal carcinoma cell lines. From at least three independent experiments, each at least with triplicate setups, mean ± SD cell viability values were calculated in percentage of values from the corresponding controls (vehicle-treated cells). Treatments (24-h incubations): ABT-737 (inhibitor of anti-apoptotic Bcl-2 family proteins; 10 μM), BEZ235 (PI3K/mTOR inhibitor; 0.10 μM), both compounds in combination. Difference in values from cells given BEZ235 or the combination treatment was compared by two-tailed Student’s *t*-test. Mutation status is indicated for each cell line. (DOCX 36 kb) Additional file 3: Fig. S2. Microscopy of cellular phenotypes under hypoxia. The three colorectal carcinoma cell lines were given combo-Rx (combination of 10 μM ABT-737, an inhibitor of anti-apoptotic Bcl-2 family proteins, and 10 μM AZD8055, an mTOR inhibitor) for 72 h under hypoxia and inspected by phase-contrast microscopy at 24-h intervals. Control cells received vehicle only. Floating pycnotic cells in medium are indicated by arrows. Scale bars: 50 μm. (DOCX 1104 kb) Additional file 4: Table S4. Tyrosine Kinase PamChip® Array substrates—phosphorylation levels. The color map visualizes normalized log_2_-transformed signal intensities from kinase substrate arrays incubated with lysates from the three colorectal carcinoma cell lines treated for 24 h with ABT-737 (inhibitor of anti-apoptotic Bcl-2 family proteins; 10 μM), AZD8055 (mTOR inhibitor; 10 μM), or combo-Rx (10 μM of both compounds in combination). Red corresponds to higher and blue to lower substrate phosphorylation levels relative to levels from the corresponding control cells. ^a^ Retrieved from UniProtKB/SwissProt (http://www.uniprot.org/). ^b^ Position(s) of the tyrosine phosphorylation site(s) within the protein. ^c^ Retrieved from PathCard (http://pathcards.genecards.org/). Super-pathway definitions were ‘PI3K-AKT signaling pathway’ and ‘MAPK signaling pathway’. (DOC 536 kb)
