# Supplementary material for: Pro-survival responses to the dual inhibition of anti-apoptotic Bcl-2 family proteins and mTOR-mediated signaling in hypoxic colorectal carcinoma cells
Source: BMC Cancer. 2016 Jul 26;16:531. doi: 10.1186/s12885-016-2600-y (PMC4962454; doi:10.1186/s12885-016-2600-y)
Supplement: Additional file 1: Fig. S1. — Cell viability of hypoxic colorectal carcinoma (CRC) cell lines. a. Four CRC cell lines were treated for 24 h with the indicated concentrations of ABT-737 (inhibitor of anti-apoptotic Bcl-2 family proteins) or AZD8055 (mTOR inhibitor) under normoxic or hypoxic culture conditions. Cell viability (measured by the MTS assay) value for each condition relative to the corresponding control cell (vehicle-treated) value is shown as mean ± SD from at least three independent experiments, each plated at least in triplicate. Statistically significant changes are indicated (asterisk, p < 0.05; cross, p < 0.01; circle, p < 0.001). b. Cultures of the CRC cell line were left in the hypoxic chamber at 0.2 % O2 and harvested after the indicated time periods. Expression of the hypoxia-inducible factor type 1α (HIF-1α) and its target gene carbonic anhydrase IX (CAIX) was examined by Western blot analysis, with α-tubulin expression as loading control, and with 24 h of normoxia and 4 h of 100 μM CoCl2 exposure in normoxia to generate negative and positive biological controls for HIF-1α expression, respectively. (DOCX 369 kb) [file 12885_2016_2600_MOESM1_ESM.docx]

**Additional file 1**

**Fig. S1** Cell viability of hypoxic colorectal carcinoma (CRC) cell lines


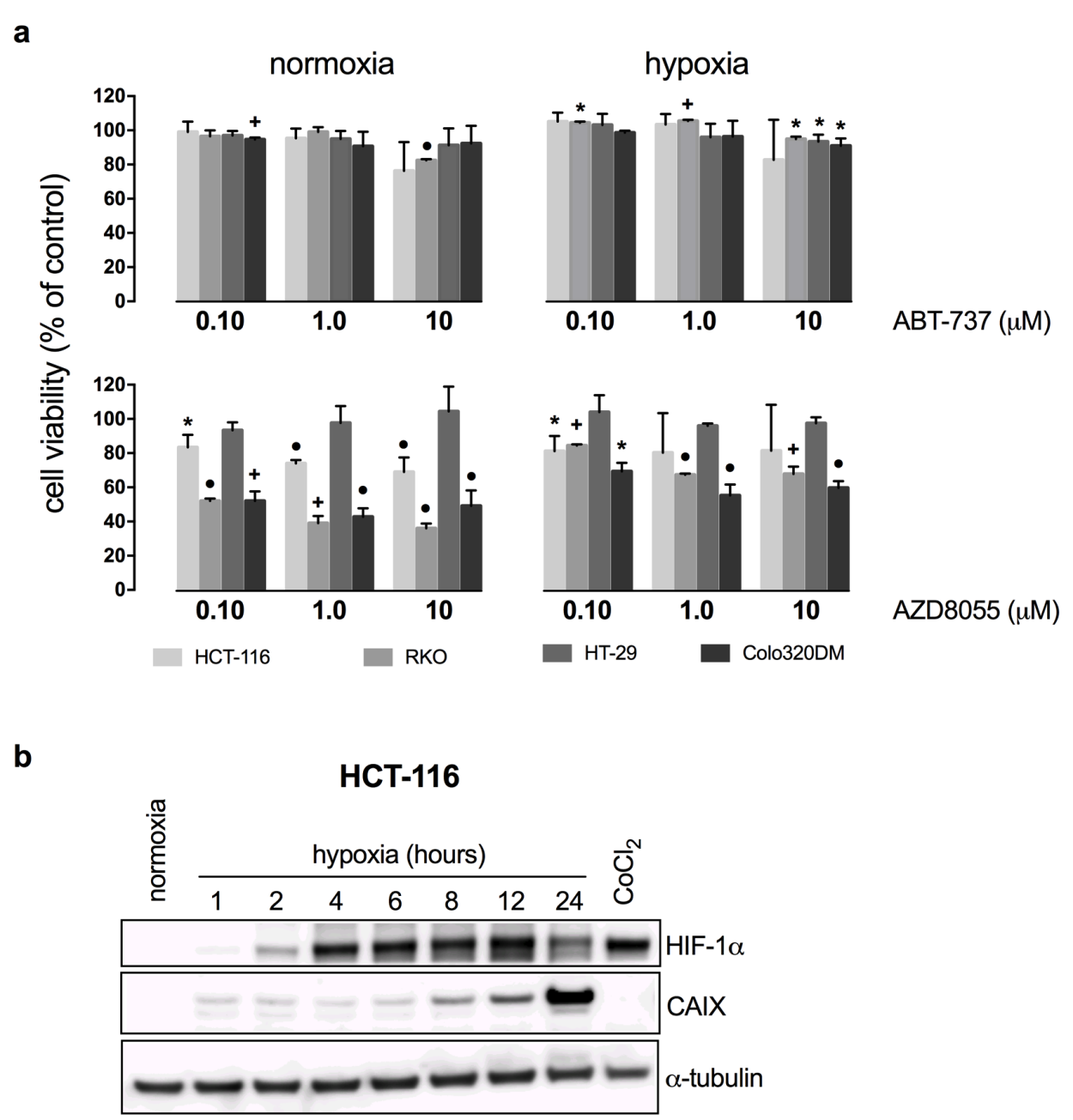


**a.** Four CRC cell lines were treated for 24 hours with the indicated concentrations of ABT-737 (inhibitor of anti-apoptotic Bcl-2 family proteins) or AZD8055 (mTOR inhibitor) under normoxic or hypoxic culture conditions. Cell viability (measured by the MTS assay) value for each condition relative to the corresponding control cell (vehicle-treated) value is shown as mean±SD from at least three independent experiments, each plated at least in triplicate. Statistically significant changes are indicated (asterisk, *p*<0.05; cross, *p*<0.01; circle, *p*<0.001). **b.** Cultures of the CRC cell line were left in the hypoxic chamber at 0.2% O_2_ and harvested after the indicated time periods. Expression of the hypoxia-inducible factor type 1α (HIF-1α) and its target gene carbonic anhydrase IX (CAIX) was examined by Western blot analysis, with α-tubulin expression as loading control, and with 24 hours of normoxia and 4 hours of 100 μM CoCl_2_ exposure in normoxia to generate negative and positive biological controls for HIF-1α expression, respectively.
