# Supplementary material for: Pro-survival responses to the dual inhibition of anti-apoptotic Bcl-2 family proteins and mTOR-mediated signaling in hypoxic colorectal carcinoma cells
Source: BMC Cancer. 2016 Jul 26;16:531. doi: 10.1186/s12885-016-2600-y (PMC4962454; doi:10.1186/s12885-016-2600-y)
Supplement: Additional file 2: Table S1a. — Cell viability of normoxic colorectal carcinoma cell lines. From at least three independent experiments, each at least with triplicate setups, mean ± SD cell viability values were calculated in percentage of values from the corresponding controls (vehicle-treated cells). Treatments (72-h incubations): ABT-737 (inhibitor of anti-apoptotic Bcl-2 family proteins; 10 μM), AZD8055 (mTOR inhibitor; 0.10 μM), both compounds in combination. Difference in values from cells given AZD8055 or the combination treatment was compared by two-tailed Student’s t-test. Mutation status is indicated for each cell line. Table S1b. Cell viability of hypoxic colorectal carcinoma cell lines. From at least three independent experiments, each at least with triplicate setups, mean ± SD cell viability values were calculated in percentage of values from the corresponding controls (vehicle-treated cells). Treatments (72-h incubations): ABT-737 (inhibitor of anti-apoptotic Bcl-2 family proteins; 10 μM), AZD8055 (mTOR inhibitor; 0.10 μM), both compounds in combination. Difference in values from cells given AZD8055 or the combination treatment was compared by two-tailed Student’s t-test. Mutation status is indicated for each cell line. Table S2. Cell viability of normoxic colorectal carcinoma cell lines. From between three and seven independent experiments, each at least with triplicate setups, mean ± SD cell viability values were calculated in percentage of values from the corresponding controls (vehicle-treated cells). Treatments (24-h incubations): ABT-737 (inhibitor of anti-apoptotic Bcl-2 family proteins; 10 μM), AZD8055 (mTOR inhibitor; 10 μM), both compounds in combination (combo-Rx). Difference in values from cells given AZD8055 or combo-Rx was compared by two-tailed Student’s t-test. Mutation status is indicated for each cell line. Table S3a. Cell viability of normoxic colorectal carcinoma cell lines. From at least three independent experiments, each at least with triplicate setups, mean ± [file 12885_2016_2600_MOESM2_ESM.docx]

**Additional file 2**

**Table S1a** Cell viability of normoxic colorectal carcinoma cell lines

| **Cell line** | **Mutations** | **ABT-737** | **AZD8055** | **Both** | ***p*-value** |
| --- | --- | --- | --- | --- | --- |
| HCT-116 | *KRAS, PIK3CA* | 94.8±7.50 | 81.5±7.85 | 82.3±5.38 | 0.88 |
| HCC2998 | *KRAS* | 35.0±2.65 | 58.0±7.81 | 18.0±3.61 | 0.0013 |
| LoVo | *KRAS* | 121±15.1 | 78.7±9.81 | 79.0±5.00 | 0.96 |
| RKO | *BRAF, PIK3CA* | 94.0±6.38 | 54.8±11.5 | 58.8±14.4 | 0.68 |
| HT-29 | *BRAF* | 74.7±9.07 | 91.7±3.79 | 58.7±16.7 | 0.029 |
| Caco-2 | wild-type | 55.8±17.0 | 37.0±9.31 | 22.8±9.84 | 0.080 |
| Colo320DM | wild-type | 99.0±1.73 | 57.3±3.06 | 51.7±13.3 | 0.51 |

From at least three independent experiments, each at least with triplicate setups, mean±SD cell viability values were calculated in percentage of values from the corresponding controls (vehicle-treated cells). Treatments (72-hours incubations): ABT-737 (inhibitor of anti-apoptotic Bcl-2 family proteins; 10 μM), AZD8055 (mTOR inhibitor; 0.10 μM), both compounds in combination. Difference in values from cells given AZD8055 or the combination treatment was compared by two-tailed Student’s *t*-test. Mutation status is indicated for each cell line.

**Table S1b** Cell viability of hypoxic colorectal carcinoma cell lines

| **Cell line** | **Mutations** | **ABT-737** | **AZD8055** | **Both** | ***p*-value** |
| --- | --- | --- | --- | --- | --- |
| HCT-116 | *KRAS, PIK3CA* | 106±11.6 | 101±10.0 | 71.7±15.0 | 0.050 |
| HCC2998 | *KRAS* | 30.7±10.8 | 73.7±11.7 | 15.7±8.08 | 0.0021 |
| LoVo | *KRAS* | 118±3.00 | 91.7±2.08 | 87.0±3.61 | 0.12 |
| RKO | *BRAF, PIK3CA* | 93.7±9.07 | 65.7±15.3 | 60.7±13.6 | 0.69 |
| HT-29 | *BRAF* | 52.0±16.4 | 97.7±6.43 | 69.7±23.3 | 0.12 |
| Caco-2 | wild-type | 41.7±17.9 | 32.3±2.08 | 17.3±6.81 | 0.022 |
| Colo320DM | wild-type | 112±4.00 | 75.7±6.03 | 59.7±4.16 | 0.019 |

From at least three independent experiments, each at least with triplicate setups, mean±SD cell viability values were calculated in percentage of values from the corresponding controls (vehicle-treated cells). Treatments (72-hours incubations): ABT-737 (inhibitor of anti-apoptotic Bcl-2 family proteins; 10 μM), AZD8055 (mTOR inhibitor; 0.10 μM), both compounds in combination. Difference in values from cells given AZD8055 or the combination treatment was compared by two-tailed Student’s *t*-test. Mutation status is indicated for each cell line.

**Table S2** Cell viability of normoxic colorectal carcinoma cell lines

| **Cell line** | **Mutations** | **ABT-737** | **AZD8055** | **Combo-Rx** | ***p*-value** |
| --- | --- | --- | --- | --- | --- |
| HCT-116 | *KRAS, PIK3CA* | 92.9±5.55 | 64.9±5.27 | 49.9±7.06 | <0.001 |
| HCT-15 | *KRAS, PIK3CA* | 92.3±3.51 | 82.0±9.85 | 69.3±7.09 | 0.14 |
| SW620 | *KRAS* | 87.8±5.74 | 56.3±7.02 | 22.3±6.66 | 0.0037 |
| SW480 | *KRAS* | 95.7±5.69 | 56.3±4.51 | 24.3±2.31 | <0.001 |
| HCC2998 | *KRAS* | 80.5±3.89 | 79.0±7.95 | 38.2±10.2 | <0.001 |
| LoVo | *KRAS* | 95.0±4.90 | 66.7±8.62 | 54.7±10.0 | 0.19 |
| RKO | *BRAF, PIK3CA* | 95.3±6.43 | 54.3±10.6 | 58.0±14.1 | 0.74 |
| KM20L2 | *BRAF* | 98.2±8.75 | 86.0±7.13 | 77.3±7.06 | 0.060 |
| WiDr | *BRAF* | 93.3±4.57 | 85.7±4.16 | 75.3±16.6 | 0.36 |
| HT-29 | *BRAF* | 96.8±6.94 | 92.7±11.9 | 83.7±9.87 | 0.37 |
| CO-115 | *BRAF* | 95.7±3.21 | 68.0±4.36 | 64.3±2.52 | 0.28 |
| Caco-2 | wild-type | 82.9±8.67 | 49.1±8.01 | 34.0±8.87 | 0.0058 |
| Colo320DM | wild-type | 95.3±5.28 | 48.0±2.28 | 48.0±2.53 | 1.0 |
| SNU-C1 | wild-type | 89.8±5.91 | 53.8±9.64 | 38.8±13.1 | 0.11 |

From between three and seven independent experiments, each at least with triplicate setups, mean±SD cell viability values were calculated in percentage of values from the corresponding controls (vehicle-treated cells). Treatments (24-hours incubations): ABT-737 (inhibitor of anti-apoptotic Bcl-2 family proteins; 10 μM), AZD8055 (mTOR inhibitor; 10 μM), both compounds in combination (combo-Rx). Difference in values from cells given AZD8055 or combo-Rx was compared by two-tailed Student’s *t*-test. Mutation status is indicated for each cell line.

**Table S3a** Cell viability of normoxic colorectal carcinoma cell lines

| **Cell line** | **Mutations** | **ABT-737** | **BEZ235** | **Both** | ***p*-value** |
| --- | --- | --- | --- | --- | --- |
| HCT-116 | *KRAS, PIK3CA* | 88.3±4.57 | 65.4±7.87 | 37.7±17.9 | 0.0027 |
| HCT-15 | *KRAS, PIK3CA* | 96.3±3.06 | 29.0±5.57 | 29.0±5.57 | 1.0 |
| SW620 | *KRAS* | 87.8±5.74 | 53.0±8.19 | 7.30±2.31 | <0.001 |
| SW480 | *KRAS* | 95.7±5.69 | 54.7±4.73 | 16.3±2.08 | <0.001 |
| HCC2998 | *KRAS* | 80.7±2.08 | 66.3±10.1 | 27.0±6.93 | 0.0051 |
| LoVo | *KRAS* | 95.0±4.90 | 49.5±6.40 | 41.0±13.8 | 0.31 |
| RKO | *BRAF, PIK3CA* | 95.3±6.43 | 35.0±11.4 | 38.7±14.6 | 0.75 |
| KM20L2 | *BRAF* | 94.8±7.98 | 73.8±4.43 | 48.8±20.4 | 0.054 |
| WiDr | *BRAF* | 93.3±4.57 | 82.0±4.36 | 45.0±11.1 | 0.0058 |
| HT-29 | *BRAF* | 96.8±6.94 | 85.5±9.52 | 46.8±6.11 | <0.001 |
| Caco-2 | wild-type | 84.5±7.77 | 55.0±11.1 | 32.0±8.89 | 0.049 |
| Colo320DM | wild-type | 95.8±5.93 | 41.3±4.57 | 38.0±6.48 | 0.44 |

From at least three independent experiments, each at least with triplicate setups, mean±SD cell viability values were calculated in percentage of values from the corresponding controls (vehicle-treated cells). Treatments (24-hours incubations): ABT-737 (inhibitor of anti-apoptotic Bcl-2 family proteins; 10 μM), BEZ235 (PI3K/mTOR inhibitor; 0.10 μM), both compounds in combination. Difference in values from cells given BEZ235 or the combination treatment was compared by two-tailed Student’s *t*-test. Mutation status is indicated for each cell line.

**Table S3b** Cell viability of hypoxic colorectal carcinoma cell lines

| **Cell line** | **Mutations** | **ABT-737** | **BEZ235** | **Both** | ***p*-value** |
| --- | --- | --- | --- | --- | --- |
| HCT-116 | *KRAS, PIK3CA* | 99.2±15.3 | 79.3±8.33 | 41.0±9.54 | 0.0063 |
| HCT-15 | *KRAS, PIK3CA* | 101±8.54 | 81.0±6.56 | 51.3±25.6 | 0.12 |
| SW620 | *KRAS* | 80.6±8.91 | 73.5±19.6 | 12.0±2.16 | <0.001 |
| SW480 | *KRAS* | 99.0±3.46 | 77.7±21.0 | 30.0±5.57 | 0.019 |
| HCC2998 | *KRAS* | 85.3±1.53 | 83.7±3.21 | 24.7±6.81 | <0.001 |
| LoVo | *KRAS* | 101±10.3 | 62.6±7.50 | 59.8±14.1 | 0.70 |
| RKO | *BRAF, PIK3CA* | 95.0±12.8 | 67.3±7.51 | 67.3±7.51 | 1.0 |
| KM20L2 | *BRAF* | 99.2±5.45 | 94.0±22.7 | 86.3±5.25 | 0.53 |
| WiDr | *BRAF* | 98.3±1.71 | 96.5±9.04 | 56.3±15.7 | 0.0044 |
| HT-29 | *BRAF* | 97.3±7.48 | 87.4±10.4 | 49.6±14.9 | <0.001 |
| Caco-2 | wild-type | 97.8±10.1 | 69.2±7.68 | 53.0±14.0 | 0.037 |
| Colo320DM | wild-type | 104±16.8 | 52.7±14.2 | 47.3±7.20 | 0.43 |

From at least three independent experiments, each at least with triplicate setups, mean±SD cell viability values were calculated in percentage of values from the corresponding controls (vehicle-treated cells). Treatments (24-hours incubations): ABT-737 (inhibitor of anti-apoptotic Bcl-2 family proteins; 10 μM), BEZ235 (PI3K/mTOR inhibitor; 0.10 μM), both compounds in combination. Difference in values from cells given BEZ235 or the combination treatment was compared by two-tailed Student’s *t*-test. Mutation status is indicated for each cell line.
