# Supplementary material for: Pro-survival responses to the dual inhibition of anti-apoptotic Bcl-2 family proteins and mTOR-mediated signaling in hypoxic colorectal carcinoma cells
Source: BMC Cancer. 2016 Jul 26;16:531. doi: 10.1186/s12885-016-2600-y (PMC4962454; doi:10.1186/s12885-016-2600-y)
Supplement: Additional file 3: Fig. S2. — Microscopy of cellular phenotypes under hypoxia. The three colorectal carcinoma cell lines were given combo-Rx (combination of 10 μM ABT-737, an inhibitor of anti-apoptotic Bcl-2 family proteins, and 10 μM AZD8055, an mTOR inhibitor) for 72 h under hypoxia and inspected by phase-contrast microscopy at 24-h intervals. Control cells received vehicle only. Floating pycnotic cells in medium are indicated by arrows. Scale bars: 50 μm. (DOCX 1104 kb) [file 12885_2016_2600_MOESM3_ESM.docx]

**Additional file 3**

**Fig. S2** Microscopy of cellular phenotypes under hypoxia


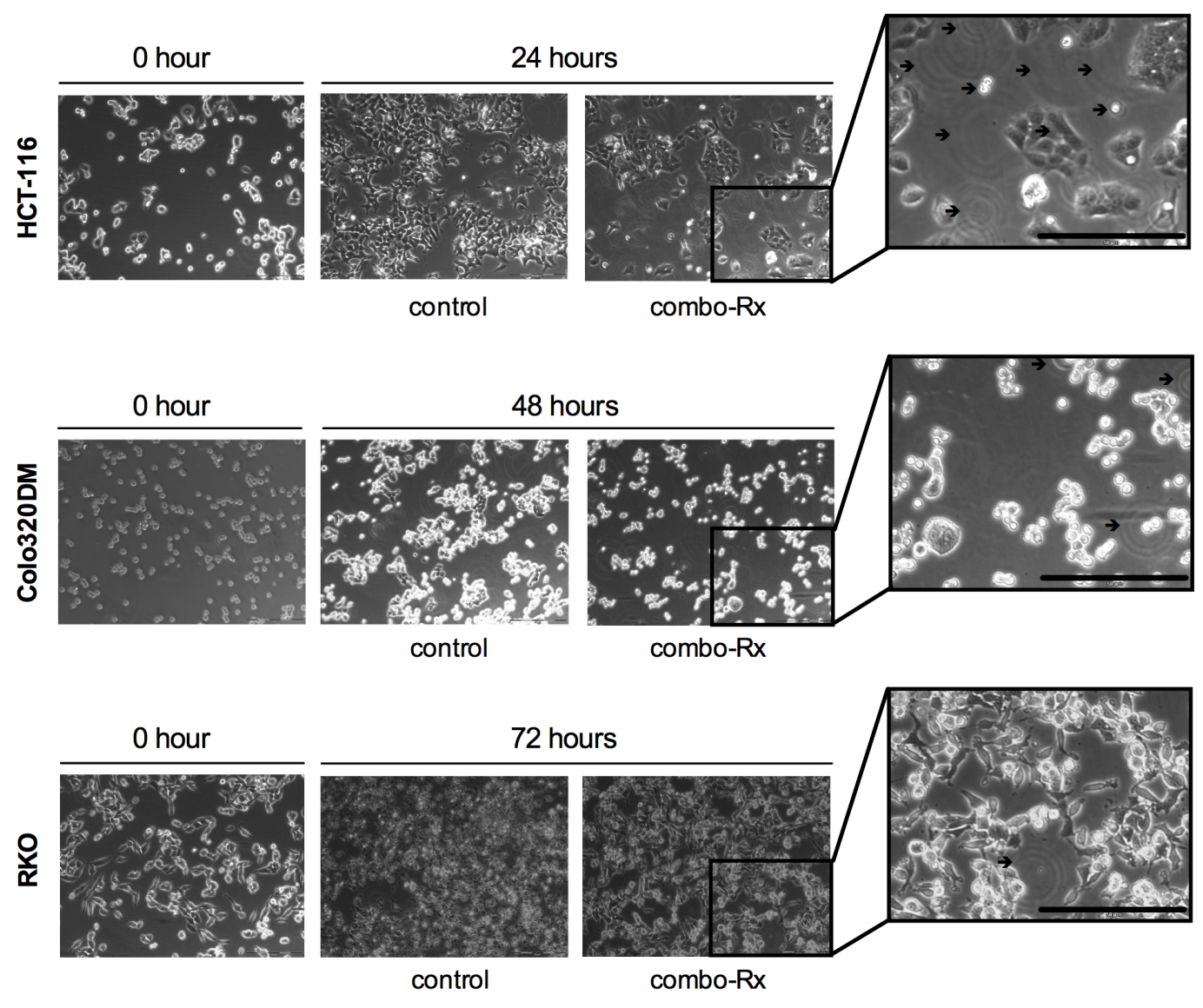


The three colorectal carcinoma cell lines were given combo-Rx (combination of 10 μM ABT-737, an inhibitor of anti-apoptotic Bcl-2 family proteins, and 10 μM AZD8055, an mTOR inhibitor) for 72 hours under hypoxia and inspected by phase-contrast microscopy at 24-hours intervals. Control cells received vehicle only. Floating pycnotic cells in medium are indicated by arrows. Scale bars: 50 μm.
