# Supplementary material for: Pro-survival responses to the dual inhibition of anti-apoptotic Bcl-2 family proteins and mTOR-mediated signaling in hypoxic colorectal carcinoma cells
Source: BMC Cancer. 2016 Jul 26;16:531. doi: 10.1186/s12885-016-2600-y (PMC4962454; doi:10.1186/s12885-016-2600-y)
Supplement: Additional file 4: Table S4. — Tyrosine Kinase PamChip® Array substrates—phosphorylation levels. The color map visualizes normalized log2-transformed signal intensities from kinase substrate arrays incubated with lysates from the three colorectal carcinoma cell lines treated for 24 h with ABT-737 (inhibitor of anti-apoptotic Bcl-2 family proteins; 10 μM), AZD8055 (mTOR inhibitor; 10 μM), or combo-Rx (10 μM of both compounds in combination). Red corresponds to higher and blue to lower substrate phosphorylation levels relative to levels from the corresponding control cells. a Retrieved from UniProtKB/SwissProt (http://www.uniprot.org/). b Position(s) of the tyrosine phosphorylation site(s) within the protein. c Retrieved from PathCard (http://pathcards.genecards.org/). Super-pathway definitions were ‘PI3K-AKT signaling pathway’ and ‘MAPK signaling pathway’. (DOC 536 kb) [file 12885_2016_2600_MOESM4_ESM.doc]

**Additional file 4**

**Table S4** Tyrosine Kinase PamChip® Array substrates – phosphorylation levels

|  |  |  | HCT-116 | | | | | | HCC2998 | | | | | | Colo320DM | | | | | |  | |  |
| --- | --- | --- | --- | --- | --- | --- | --- | --- | --- | --- | --- | --- | --- | --- | --- | --- | --- | --- | --- | --- | --- | --- | --- |
|  |  |  |  | | | | | |  | | | | | |  | | | | | |  | |  |
|  |  |  |  |  |  |  |  |  |  |  |  |  |  |  |  |  |  |  |  |  | |  | |
|  |  |  | normoxia | | | hypoxia | | | normoxia | | | hypoxia | | | normoxia | | | hypoxia | | | |  | |
|  |  |  |  |  |  |  |  |  |  |  |  |  |  |  |  |  |  |  |  |  | |  | |
|  |  |  | ABT-737 | AZD8055 | combo-Rx | ABT-737 | AZD8055 | combo-Rx | ABT-737 | AZD8055 | combo-Rx | ABT-737 | AZD8055 | combo-Rx | ABT-737 | AZD8055 | combo-Rx | ABT-737 | AZD8055 | combo-Rx | |  | |
| **Gene name^a^** | **Peptide sequence** | **Tyrosine position^b^** |  |  |  |  |  |  |  |  |  |  |  |  |  |  |  |  |  |  | | **Pathway^c^** | |
| CDK2 | EKIGEGTYGVVYK | [15, 19] |  |  |  |  |  |  |  |  |  |  |  |  |  |  |  |  |  |  | | PI3K-AKT | |
| EGFR | EDSFLQRYSSDPT | [1069] |  |  |  |  |  |  |  |  |  |  |  |  |  |  |  |  |  |  | | PI3K-AKT | |
| EGFR | GSVQNPVYHNQPL | [1110] |  |  |  |  |  |  |  |  |  |  |  |  |  |  |  |  |  |  | | PI3K-AKT | |
| EGFR | APSRDPHYQDPHS | [1125] |  |  |  |  |  |  |  |  |  |  |  |  |  |  |  |  |  |  | | PI3K-AKT | |
| EGFR | ISLDNPDYQQDFF | [1172] |  |  |  |  |  |  |  |  |  |  |  |  |  |  |  |  |  |  | | PI3K-AKT | |
| EGFR | STAENAEYLRVAP | [1197] |  |  |  |  |  |  |  |  |  |  |  |  |  |  |  |  |  |  | | PI3K-AKT | |
| EGFR | LGAEEKEYHAEGG | [869] |  |  |  |  |  |  |  |  |  |  |  |  |  |  |  |  |  |  | | PI3K-AKT | |
| EGFR | MTFGSKPYDGIPA | [915] |  |  |  |  |  |  |  |  |  |  |  |  |  |  |  |  |  |  | | PI3K-AKT | |
| EPHA2 | QLKPLKTYVDPHT | [588] |  |  |  |  |  |  |  |  |  |  |  |  |  |  |  |  |  |  | | PI3K-AKT | |
| EPHA2 | EDDPEATYTTSGG | [772] |  |  |  |  |  |  |  |  |  |  |  |  |  |  |  |  |  |  | | PI3K-AKT | |
| EPOR | SEHAQDTYLVLDK | [368] |  |  |  |  |  |  |  |  |  |  |  |  |  |  |  |  |  |  | | PI3K-AKT | |
| EPOR | ASAASFEYTILDP | [426] |  |  |  |  |  |  |  |  |  |  |  |  |  |  |  |  |  |  | | PI3K-AKT | |
| PTK2B | RYMEDSTYYKASK | [570, 576, 577] |  |  |  |  |  |  |  |  |  |  |  |  |  |  |  |  |  |  | | PI3K-AKT | |
| PTK2B | RYIEDEDYYKASV | [573, 579, 580] |  |  |  |  |  |  |  |  |  |  |  |  |  |  |  |  |  |  | | PI3K-AKT | |
| INSR | SLGFKRSYEEHIP | [992, 999] |  |  |  |  |  |  |  |  |  |  |  |  |  |  |  |  |  |  | | PI3K-AKT | |
| INSR | YASSNPEYLSASD | [1355] |  |  |  |  |  |  |  |  |  |  |  |  |  |  |  |  |  |  | | PI3K-AKT | |
| JAK1 | AIETDKEYYTVKD | [1022, 1023] |  |  |  |  |  |  |  |  |  |  |  |  |  |  |  |  |  |  | | PI3K-AKT | |
| JAK2 | VRREVGDYGQLHETE | [570] |  |  |  |  |  |  |  |  |  |  |  |  |  |  |  |  |  |  | | PI3K-AKT | |
| SYK | ALRADENYYKAQT | [525, 526] |  |  |  |  |  |  |  |  |  |  |  |  |  |  |  |  |  |  | | PI3K-AKT | |
| PDPK1 | ARTTSQLYDAVPI | [9] |  |  |  |  |  |  |  |  |  |  |  |  |  |  |  |  |  |  | | PI3K-AKT | |
| PDPK1 | DEDCYGNYDNLLS | [373, 376] |  |  |  |  |  |  |  |  |  |  |  |  |  |  |  |  |  |  | | PI3K-AKT | |
| PPP2CB | EPHVTRRTPDYFL | [307] |  |  |  |  |  |  |  |  |  |  |  |  |  |  |  |  |  |  | | PI3K-AKT | |
| RBL2 | VPTVSKGTVEGNY | [111] |  |  |  |  |  |  |  |  |  |  |  |  |  |  |  |  |  |  | | PI3K-AKT | |
| ERBB2 | PTAENPEYLGLDV | [1248] |  |  |  |  |  |  |  |  |  |  |  |  |  |  |  |  |  |  | | MAPK | |
| ERBB2 | LDIDETEYHADGG | [877] |  |  |  |  |  |  |  |  |  |  |  |  |  |  |  |  |  |  | | MAPK | |
| ERBB4 | QALDNPEYHNASN | [1188] |  |  |  |  |  |  |  |  |  |  |  |  |  |  |  |  |  |  | | MAPK | |
| ERBB4 | IVAENPEYLSEFS | [1284] |  |  |  |  |  |  |  |  |  |  |  |  |  |  |  |  |  |  | | MAPK | |
| MAPK7 | AEHQYFMTEYVAT | [215, 220] |  |  |  |  |  |  |  |  |  |  |  |  |  |  |  |  |  |  | | MAPK | |
| MAPK10 | TSFMMTPYVVTRY | [223, 228] |  |  |  |  |  |  |  |  |  |  |  |  |  |  |  |  |  |  | | MAPK | |
| MAPK12 | ADSEMTGYVVTRW | [185] |  |  |  |  |  |  |  |  |  |  |  |  |  |  |  |  |  |  | | MAPK | |
| MAPK14 | RHTDDEMTGYVAT | [182] |  |  |  |  |  |  |  |  |  |  |  |  |  |  |  |  |  |  | | MAPK | |
| NTRK1 | HIIENPQYFSDAC | [496] |  |  |  |  |  |  |  |  |  |  |  |  |  |  |  |  |  |  | | MAPK | |
| NTRK2 | PVIENPQYFGITN | [516] |  |  |  |  |  |  |  |  |  |  |  |  |  |  |  |  |  |  | | MAPK | |
| NTRK2 | GMSRDVYSTDYYR | [702, 706, 707] |  |  |  |  |  |  |  |  |  |  |  |  |  |  |  |  |  |  | | MAPK | |
| RASA1 | TVDGKEIYNTIRR | [460] |  |  |  |  |  |  |  |  |  |  |  |  |  |  |  |  |  |  | | MAPK | |
| STAT1 | DGPKGTGYIKTEL | [701] |  |  |  |  |  |  |  |  |  |  |  |  |  |  |  |  |  |  | | MAPK | |
| STAT3 | DPGSAAPYLKTKF | [705] |  |  |  |  |  |  |  |  |  |  |  |  |  |  |  |  |  |  | | MAPK | |
| FGFR1 | TSNQEYLDLSMPL | [766] |  |  |  |  |  |  |  |  |  |  |  |  |  |  |  |  |  |  | | PI3K-AKT and MAPK | |
| FGFR2 | TLTTNEEYLDLSQ | [769] |  |  |  |  |  |  |  |  |  |  |  |  |  |  |  |  |  |  | | PI3K-AKT and MAPK | |
| FGFR3 | DVHNLDYYKKTTN | [647, 648] |  |  |  |  |  |  |  |  |  |  |  |  |  |  |  |  |  |  | | PI3K-AKT and MAPK | |
| FGFR3 | TVTSTDEYLDLSA | [760] |  |  |  |  |  |  |  |  |  |  |  |  |  |  |  |  |  |  | | PI3K-AKT and MAPK | |
| MAPK1 | HTGFLTEYVATRW | [187] |  |  |  |  |  |  |  |  |  |  |  |  |  |  |  |  |  |  | | PI3K-AKT and MAPK | |
| MAPK1 | IMLNSKGYTKSID | [205] |  |  |  |  |  |  |  |  |  |  |  |  |  |  |  |  |  |  | | PI3K-AKT and MAPK | |
| PIK3R1 | NENTEDQYSLVED | [607] |  |  |  |  |  |  |  |  |  |  |  |  |  |  |  |  |  |  | | PI3K-AKT and MAPK | |
| PDGFRB | LDTSSVLYTAVQP | [1009] |  |  |  |  |  |  |  |  |  |  |  |  |  |  |  |  |  |  | | PI3K-AKT and MAPK | |
| PDGFRB | PNEGDNDYIIPLPDP | [1021] |  |  |  |  |  |  |  |  |  |  |  |  |  |  |  |  |  |  | | PI3K-AKT and MAPK | |
| PDGFRB | VSSDGHEYIYVDP | [579, 581] |  |  |  |  |  |  |  |  |  |  |  |  |  |  |  |  |  |  | | PI3K-AKT and MAPK | |
| PDGFRB | RPPSAELYSNALP | [716] |  |  |  |  |  |  |  |  |  |  |  |  |  |  |  |  |  |  | | PI3K-AKT and MAPK | |
| PDGFRB | SSNYMAPYDNYVP | [771, 775, 778] |  |  |  |  |  |  |  |  |  |  |  |  |  |  |  |  |  |  | | PI3K-AKT and MAPK | |
| PDGFRB | YMAPYDNYVPSAP | [771, 775, 778] |  |  |  |  |  |  |  |  |  |  |  |  |  |  |  |  |  |  | | PI3K-AKT and MAPK | |
| RAF1 | PRGQRDSSYYWEI | [340, 341] |  |  |  |  |  |  |  |  |  |  |  |  |  |  |  |  |  |  | | PI3K-AKT and MAPK | |
| EBP41 | LDGENIYIRHSNL | [660] |  |  |  |  |  |  |  |  |  |  |  |  |  |  |  |  |  |  | | Other | |
| CHRND | YISKAEEYFLLKS | [383, 390] |  |  |  |  |  |  |  |  |  |  |  |  |  |  |  |  |  |  | | Other | |
| ENPEP | EREGSKRYCIQTK | [12] |  |  |  |  |  |  |  |  |  |  |  |  |  |  |  |  |  |  | | Other | |
| ANXA1 | IENEEQEYVQTVK | [21] |  |  |  |  |  |  |  |  |  |  |  |  |  |  |  |  |  |  | | Other | |
| ANXA2 | HSTPPSAYGSVKA | [24] |  |  |  |  |  |  |  |  |  |  |  |  |  |  |  |  |  |  | | Other | |
| SLC4A1 | TEATATDYHTTSH | [46] |  |  |  |  |  |  |  |  |  |  |  |  |  |  |  |  |  |  | | Other | |
| C1R | TEASGYISSLEYP | [204, 210] |  |  |  |  |  |  |  |  |  |  |  |  |  |  |  |  |  |  | | Other | |
| CALM1 | FDKDGNGYISAAE | [100] |  |  |  |  |  |  |  |  |  |  |  |  |  |  |  |  |  |  | | Other | |
| CALM1 | KDGNGYISAAELR | [100] |  |  |  |  |  |  |  |  |  |  |  |  |  |  |  |  |  |  | | Other | |
| CBL | EGEEDTEYMTPSS | [700] |  |  |  |  |  |  |  |  |  |  |  |  |  |  |  |  |  |  | | Other | |
| CD247 | KDKMAEAYSEIGM | [123] |  |  |  |  |  |  |  |  |  |  |  |  |  |  |  |  |  |  | | Other | |
| CD247 | STATKDTYDALHM | [153] |  |  |  |  |  |  |  |  |  |  |  |  |  |  |  |  |  |  | | Other | |
| CD79A | EYEDENLYEGLNL | [182, 188] |  |  |  |  |  |  |  |  |  |  |  |  |  |  |  |  |  |  | | Other | |
| CDK7 | GLAKSFGSPNRAY | [169] |  |  |  |  |  |  |  |  |  |  |  |  |  |  |  |  |  |  | | Other | |
| CRK | GPPEPGPYAQPSV | [221] |  |  |  |  |  |  |  |  |  |  |  |  |  |  |  |  |  |  | | Other | |
| CTNNB1 | VADIDGQYAMTRA | [86] |  |  |  |  |  |  |  |  |  |  |  |  |  |  |  |  |  |  | | Other | |
| DCX | GIVYAVSSDRFRS | [112] |  |  |  |  |  |  |  |  |  |  |  |  |  |  |  |  |  |  | | Other | |
| DDR1 | LLLSNPAYRLLLA | [513] |  |  |  |  |  |  |  |  |  |  |  |  |  |  |  |  |  |  | | Other | |
| DYRK1A | KHDTEMKYYIVHL | [219, 220] |  |  |  |  |  |  |  |  |  |  |  |  |  |  |  |  |  |  | | Other | |
| DYRK1A | CQLGQRIYQYIQS | [319, 321] |  |  |  |  |  |  |  |  |  |  |  |  |  |  |  |  |  |  | | Other | |
| EFS | GGTDEGIYDVPLL | [253] |  |  |  |  |  |  |  |  |  |  |  |  |  |  |  |  |  |  | | Other | |
| EFS | GGTDEGIFDVPLL | NA |  |  |  |  |  |  |  |  |  |  |  |  |  |  |  |  |  |  | | Other | |
| ENO2 | SGASTGIYEALEL | [44] |  |  |  |  |  |  |  |  |  |  |  |  |  |  |  |  |  |  | | Other | |
| EPHA1 | LDDFDGTYETQGG | [781] |  |  |  |  |  |  |  |  |  |  |  |  |  |  |  |  |  |  | | Other | |
| EPHA4 | LNQGVRTYVDPFT | [596] |  |  |  |  |  |  |  |  |  |  |  |  |  |  |  |  |  |  | | Other | |
| EPHA4 | QAIKMDRYKDNFT | [928] |  |  |  |  |  |  |  |  |  |  |  |  |  |  |  |  |  |  | | Other | |
| EPHA7 | TYIDPETYEDPNR | [608, 614] |  |  |  |  |  |  |  |  |  |  |  |  |  |  |  |  |  |  | | Other | |
| EPHB1 | DDTSDPTYTSSLG | [778] |  |  |  |  |  |  |  |  |  |  |  |  |  |  |  |  |  |  | | Other | |
| EPHB1 | SAIKMVQYRDSFL | [928] |  |  |  |  |  |  |  |  |  |  |  |  |  |  |  |  |  |  | | Other | |
| EPHB4 | IGHGTKVYIDPFT | [590] |  |  |  |  |  |  |  |  |  |  |  |  |  |  |  |  |  |  | | Other | |
| FABP3 | DSKNFDDYMKSLG | [20] |  |  |  |  |  |  |  |  |  |  |  |  |  |  |  |  |  |  | | Other | |
| FER | RQEDGGVYSSSGL | [714] |  |  |  |  |  |  |  |  |  |  |  |  |  |  |  |  |  |  | | Other | |
| FES | REEADGVYAASGG | [713] |  |  |  |  |  |  |  |  |  |  |  |  |  |  |  |  |  |  | | Other | |
| FRK | KVDNEDIYESRHE | [387] |  |  |  |  |  |  |  |  |  |  |  |  |  |  |  |  |  |  | | Other | |
| KRT6B | GAGFGSRSLYGLG | [62] |  |  |  |  |  |  |  |  |  |  |  |  |  |  |  |  |  |  | | Other | |
| KRT8 | SAYGGLTSPGLSY | [427, 437] |  |  |  |  |  |  |  |  |  |  |  |  |  |  |  |  |  |  | | Other | |
| LAT | MESIDDYVNVPES | [200] |  |  |  |  |  |  |  |  |  |  |  |  |  |  |  |  |  |  | | Other | |
| LAT | EEGAPDYENLQEL | [255] |  |  |  |  |  |  |  |  |  |  |  |  |  |  |  |  |  |  | | Other | |
| LCK | RLIEDNEYTAREG | [394] |  |  |  |  |  |  |  |  |  |  |  |  |  |  |  |  |  |  | | Other | |
| MBP | ARTAHYGSLPQKS | [203] |  |  |  |  |  |  |  |  |  |  |  |  |  |  |  |  |  |  | | Other | |
| MBP | FGYGGRASDYKSA | [261, 268] |  |  |  |  |  |  |  |  |  |  |  |  |  |  |  |  |  |  | | Other | |
| MBP | GRASDYKSAHKGF | [268] |  |  |  |  |  |  |  |  |  |  |  |  |  |  |  |  |  |  | | Other | |
| MET | RDMYDKEYYSVHN | [1230, 1234, 1235] |  |  |  |  |  |  |  |  |  |  |  |  |  |  |  |  |  |  | | Other | |
| NCF1 | QRSRKRLSQDAYR | [324] |  |  |  |  |  |  |  |  |  |  |  |  |  |  |  |  |  |  | | Other | |
| SLC34A1 | AKALGKRTAKYRW | [511] |  |  |  |  |  |  |  |  |  |  |  |  |  |  |  |  |  |  | | Other | |
| BCKDHA | DDSSAYRSVDEVN | [345] |  |  |  |  |  |  |  |  |  |  |  |  |  |  |  |  |  |  | | Other | |
| PDHA1 | SMSDPGVSYRTRE | [299] |  |  |  |  |  |  |  |  |  |  |  |  |  |  |  |  |  |  | | Other | |
| PXN | VGEEEHVYSFPNK | [118] |  |  |  |  |  |  |  |  |  |  |  |  |  |  |  |  |  |  | | Other | |
| PXN | FLSEETPYSYPTG | [31, 33] |  |  |  |  |  |  |  |  |  |  |  |  |  |  |  |  |  |  | | Other | |
| PECAM1 | KKDTETVYSEVRK | [713] |  |  |  |  |  |  |  |  |  |  |  |  |  |  |  |  |  |  | | Other | |
| PRPH | QRSELDKSSAHSY | [470] |  |  |  |  |  |  |  |  |  |  |  |  |  |  |  |  |  |  | | Other | |
| PLCG1 | EGSFESRYQQPFE | [1253] |  |  |  |  |  |  |  |  |  |  |  |  |  |  |  |  |  |  | | Other | |
| PLCG1 | IGTAEPDYGALYE | [771, 775] |  |  |  |  |  |  |  |  |  |  |  |  |  |  |  |  |  |  | | Other | |
| PLCG1 | EGRNPGFYVEANP | [783] |  |  |  |  |  |  |  |  |  |  |  |  |  |  |  |  |  |  | | Other | |
| PGR | LRPDSEASQSPQY | [557] |  |  |  |  |  |  |  |  |  |  |  |  |  |  |  |  |  |  | | Other | |
| PGR | EQRMKESSFYSLC | [795] |  |  |  |  |  |  |  |  |  |  |  |  |  |  |  |  |  |  | | Other | |
| PRRX2 | WTASSPYSTVPPY | [208, 214] |  |  |  |  |  |  |  |  |  |  |  |  |  |  |  |  |  |  | | Other | |
| PTPN11 | SKRKGHEYTNIKY | [546, 551] |  |  |  |  |  |  |  |  |  |  |  |  |  |  |  |  |  |  | | Other | |
| RB1 | IYISPLKSPYKIS | [805, 813] |  |  |  |  |  |  |  |  |  |  |  |  |  |  |  |  |  |  | | Other | |
| RET | TPSDSLIYDDGLS | [1029] |  |  |  |  |  |  |  |  |  |  |  |  |  |  |  |  |  |  | | Other | |
| RET | AQAFPVSYSSSGA | [687] |  |  |  |  |  |  |  |  |  |  |  |  |  |  |  |  |  |  | | Other | |
| MST1R | SALLGDHYVQLPA | [1353] |  |  |  |  |  |  |  |  |  |  |  |  |  |  |  |  |  |  | | Other | |
| MST1R | YVQLPATYMNLGP | [1353, 1360] |  |  |  |  |  |  |  |  |  |  |  |  |  |  |  |  |  |  | | Other | |
| CTTN1 | VSQREAEYEPETV | [477] |  |  |  |  |  |  |  |  |  |  |  |  |  |  |  |  |  |  | | Other | |
| CTTN1 | EYEPETVYEVAGA | [477, 483] |  |  |  |  |  |  |  |  |  |  |  |  |  |  |  |  |  |  | | Other | |
| CTTN1 | YQAEENTYDEYEN | [492, 499, 502] |  |  |  |  |  |  |  |  |  |  |  |  |  |  |  |  |  |  | | Other | |
| STAT5A | LAKAVDGYVKPQI | [694] |  |  |  |  |  |  |  |  |  |  |  |  |  |  |  |  |  |  | | Other | |
| STAT4 | TERGDKGYVPSVF | [693] |  |  |  |  |  |  |  |  |  |  |  |  |  |  |  |  |  |  | | Other | |
| STAT4 | PSDLLPMSPSVYA | [725] |  |  |  |  |  |  |  |  |  |  |  |  |  |  |  |  |  |  | | Other | |
| STAT6 | MGKDGRGYVPATI | [641] |  |  |  |  |  |  |  |  |  |  |  |  |  |  |  |  |  |  | | Other | |
| TEC | RYFLDDQYTSSSG | [513, 519] |  |  |  |  |  |  |  |  |  |  |  |  |  |  |  |  |  |  | | Other | |
| TNNT1 | SDTEEQEYEEEQP | [9] |  |  |  |  |  |  |  |  |  |  |  |  |  |  |  |  |  |  | | Other | |
| TYRO3 | KIYSGDYYRQGCA | [681, 685, 686] |  |  |  |  |  |  |  |  |  |  |  |  |  |  |  |  |  |  | | Other | |
| FLT1 | DFGLARDIYKNPD | [1048] |  |  |  |  |  |  |  |  |  |  |  |  |  |  |  |  |  |  | | Other | |
| FLT1 | DIFKNPDYVRKGD | [1053] |  |  |  |  |  |  |  |  |  |  |  |  |  |  |  |  |  |  | | Other | |
| FLT1 | KNPDYVRKGDTRL | [1053] |  |  |  |  |  |  |  |  |  |  |  |  |  |  |  |  |  |  | | Other | |
| FLT1 | VQQDGKDYIPINA | [1169] |  |  |  |  |  |  |  |  |  |  |  |  |  |  |  |  |  |  | | Other | |
| FLT1 | GSSDDVRYVNAFK | [1213] |  |  |  |  |  |  |  |  |  |  |  |  |  |  |  |  |  |  | | Other | |
| FLT1 | ATSMFDDYQGDSS | [1242] |  |  |  |  |  |  |  |  |  |  |  |  |  |  |  |  |  |  | | Other | |
| FLT1 | SSSPPPDYNSVVL | [1327] |  |  |  |  |  |  |  |  |  |  |  |  |  |  |  |  |  |  | | Other | |
| FLT1 | DYNSVVLYSTPPI | [1327, 1333] |  |  |  |  |  |  |  |  |  |  |  |  |  |  |  |  |  |  | | Other | |
| KDR | RFRQGKDYVGAIP | [951] |  |  |  |  |  |  |  |  |  |  |  |  |  |  |  |  |  |  | | Other | |
| KDR | EEAPEDLYKDFLT | [996] |  |  |  |  |  |  |  |  |  |  |  |  |  |  |  |  |  |  | | Other | |
| KDR | DFGLARDIYKDPD | [1054] |  |  |  |  |  |  |  |  |  |  |  |  |  |  |  |  |  |  | | Other | |
| KDR | DIYKDPDYVRKGD | [1054, 1059] |  |  |  |  |  |  |  |  |  |  |  |  |  |  |  |  |  |  | | Other | |
| KDR | AQQDGKDYIVLPI | [1175] |  |  |  |  |  |  |  |  |  |  |  |  |  |  |  |  |  |  | | Other | |
| KDR | VSDPKFHYDNTAG | [1214] |  |  |  |  |  |  |  |  |  |  |  |  |  |  |  |  |  |  | | Other | |
| FLT4 | DIYKDPDYVRKGS | [1063, 1068] |  |  |  |  |  |  |  |  |  |  |  |  |  |  |  |  |  |  | | Other | |
| VCL | KSFLDSGYRILGA | [822] |  |  |  |  |  |  |  |  |  |  |  |  |  |  |  |  |  |  | | Other | |
| ZAP70 | ALGADDSYYTARS | [492, 493] |  |  |  |  |  |  |  |  |  |  |  |  |  |  |  |  |  |  | | Other | |
| ZBTB16 | LRTHNGASPYQCT | [630] |  |  |  |  |  |  |  |  |  |  |  |  |  |  |  |  |  |  | | Other | |

The color map visualizes normalized log_2_-transformed signal intensities from kinase substrate arrays incubated with lysates from the three colorectal carcinoma cell lines treated for 24 hours with ABT-737 (inhibitor of anti-apoptotic Bcl-2 family proteins; 10 μM), AZD8055 (mTOR inhibitor; 10 μM), or combo-Rx (10 μM of both compounds in combination). Red corresponds to higher and blue to lower substrate phosphorylation levels relative to levels from the corresponding control cells.

^a^ Retrieved from UniProtKB/SwissProt ([www.uniprot.org](http://www.uniprot.org/)).

^b^ Position(s) of the tyrosine phosphorylation site(s) within the protein.

^c^ Retrieved from PathCard ([www.pathcards.genecards.org](http://www.pathcards.genecards.org/)). Super-pathway definitions were ‘PI3K-AKT signaling pathway’ and ‘MAPK signaling pathway’.

| Log_2_ fold-change: | | |
| --- | --- | --- |
| -0.50 or lower |  |  |
| -0.50 to 0 |  |  |
| 0 to 0.50 |  |  |
| 0.50 to 1.0 |  |  |
| 1.0 to 1.5 |  |  |
| 1.5 to 2.0 |  |  |
| 2.0 or higher |  |  |
